# Translation, contextual adaptation, and preliminary evaluation of INCRESE-Norwegian Young

**DOI:** 10.3389/fpsyt.2026.1893622

**Published:** 2026-10-14

**Authors:** Anna Leiler, Kristin Øksendal Børresen, Malle Vogelsang, Jone Trovåg, Ingunn Anita Kulstad, Cathrine Moe, Helga Dis-Isfold Sigurdardottir, Victor Manuel Pèrez Colado, Teddy Nambazira, Fiona Ng, Stefan Rennick-Egglestone, Mike Slade, Paal William Craig Botnvik, Siv Anita Aasum, Unn Kristin Vedal, Kristin Berre Ørjasæter

**Affiliations:** 1 Faculty of Nursing and Health Sciences, Nord University, Levanger, Norway; 2 Mid Sweden University, Department of Education, Psychology and Social Work, Östersund, Sweden; 3 Faculty of Nursing and Health Sciences, Nord University, Bodø, Norway; 4 Youth Mental Health Norway, Oslo, Norway; 5 Nord University, Faculty of Social Sciences, Levanger, Norway; 6 School of Health Sciences, Institute of Mental Health, University of Nottingham, Nottingham, United Kingdom; 7 National Institute for Health and Care Research (NIHR) Nottingham Biomedical Research Centre, Institute of Mental Health, Nottingham, United Kingdom; 8 Mental Health and Substance Abuse Services, Snåsa, Norway; 9 Faculty of Nursing and Health Sciences, Nord University, Namsos, Norway

**Keywords:** contextual adaptation, inventory, mental health, recovery narratives, recovery stories, reliability, translation, young people

## Abstract

**Background:**

The mental health of young people is a global concern. Norway has seen a rise in mental health challenges among young individuals in recent decades, including both Sámi and non-Sámi youth. Culturally responsive and accessible mental health interventions are needed. The NEON Young Norway Study investigates the use of mental health recovery narratives as a low threshold intervention. To engage in recovery narratives mostly have beneficial impact, however, there is also a risk of triggering harmful behavior, which is why safe use of narratives requires careful analysis. For this reason, the Inventory of the Characteristics of REcovery StoriEs (INCRESE) has been developed in English. Our aim was to translate, contextually adapt, and to preliminary evaluate INCRESE for use among young people in Norway.

**Methods:**

INCRESE was translated into Norwegian using the UPSIDES proportionate translation methodology. Contextual adaptation was carried out in collaboration with cultural experts and young individuals with lived experience. For reliability assessment, five coders independently coded 32 narratives (whereof 6 Sámi) twice, with a two-week interval between assessments. Test-retest reliability was assessed with Cohen’s kappa and interrater reliability with Gwet’s AC1, with scores above 0.40 considered acceptable. Feasibility and acceptability were assessed qualitatively.

**Results:**

Multiple adaptations were required to ensure contextual fit for both general Norwegian and Sámi youth. Test–retest reliability (Cohen’s K) was moderate, with a mean κ = 0.59 (SD = 0.27; range –0.02 to 1.00). Mean percentage agreement between the two rating rounds was 90.7% (SD = 10.03). Interrater reliability (Gwet’s AC1) ranged from 0.36 (fair) to 1.00 (perfect) per item, mean AC1 0.86 (SD = 0.14), and with a mean percentual agreement of 72.8% (SD = 26.64). Reliability varied considerably between items. Coders generally found INCRESE-NY feasible and acceptable and provided suggestions for further refinement.

**Conclusions:**

INCRESE-NY showed overall acceptable reliability for characterizing recovery narratives produced by young Norwegians, including Sámi youth. The adapted inventory appears promising as a tool that can support the safe and systematic use of recovery narratives in youth mental health contexts. However, further refinement is needed to examine its conceptual, cultural and practical applicability.

## 1 Introduction

Recorded Recovery Narratives, first-person accounts of living with and recovering from mental health challenges, are being used in research and interventions to foster hope, empowerment, and reduced self-stigma (1, 2). To support the systematic and responsible use of such narratives, the Inventory of the Characteristics of REcovery StoriEs (INCRESE) has been developed in English to capture key narrative features, structures, and potential impacts such as content that might create distress for some (3). However, INCRESE is largely based on recovery narratives produced by adults and majority populations, limiting its applicability to young people and indigenous groups. Youth recovery processes are shaped by distinct developmental, social, and cultural factors, and indigenous youth, such as the Norwegian Sámi, may hold experiences and forms of meaning-making that are not adequately represented in current frameworks. Consequently, there is a need for contextually and developmentally adapted tools to ensure valid and safe use of recovery narratives in youth mental health research and practice. The present study responds to this need by translating, contextually adapting, and evaluating a Norwegian version of INCRESE for use with both Sámi and non-Sámi individuals aged 16–30 in Norway.

The relevance of recovery-oriented approaches is heightened by the increasing mental health burden among young people, which has become a global concern. A recent review reports that one in five European adolescents at present experience mental health challenges (4). In Norway, analysis of data from the population-based Trøndelag Health Study (HUNT) shows a marked rise in mental health challenges such as anxiety and depression symptoms among adolescents and young adults between 1995 and 2019 (5). In the most recent decade examined in the HUNT study, nearly 45% of females aged 13–19 reported symptoms of anxiety and depression (5). The COVID-19 pandemic and associated quarantine measures placed additional strain on the mental health of some young Norwegians, and those who experienced distress during this period appear to be at heightened risk for continued challenges (6).

Within the Norwegian context, it is important to consider how youth mental health manifests across different population groups. Norway is home to the Sámi, an indigenous people of the northern parts of Scandinavia. Norwegian Sámi adolescents identify as Sámi, Norwegian or both (7). Research suggests that young Sámi’s overall mental health burden is comparable to that of non-Sámi in Norway (8), and rates of self-harm and suicidal behavior also appear similar across groups (9). However, young Sámi experience more discrimination than non-Sámi youth, and such experiences are associated with increased levels of internalizing mental health symptoms (10). At the same time, a strong Sámi ethnic identification has been identified as a protective factor for mental health (11). Together, this suggests that young Sámi may not differ in overall mental health outcomes but are exposed to distinct risk and protective processes relevant to mental health recovery.

Understanding these processes requires a recovery-oriented perspective. One way of defining mental health recovery is as “a deeply personal, unique process of changing one’s attitudes, values, feelings, goals, skills, and/or roles. It is a way of living a satisfying, hopeful, and contributing life even with limitations caused by illness” (12, p 527). Current understanding of recovery processes primarily derives from personal stories shared and recorded by individuals with lived experience of recovering from mental health challenges, herein referred to as recovery narratives (13). The definition of recovery narratives as first-person non-fiction accounts of recovery from mental health issues, including elements of adversity or struggle, and of self-defined strengths, successes, or survival is intentionally broad to encompass narratives from a wide and diverse group of people (3).

The sharing of recorded recovery narratives has become a central element of recovery-oriented interventions (14). Recovery narratives vary widely in form, structure and content (13) and engaging with them can affect recipients cognitively, emotionally and behaviorally (15). While these effects are often beneficial, such as reducing self-stigma and fostering hope, negative impacts such as triggering painful memories, sadness or communication of harmful behaviors may also arise (15).

Consequently, it is essential to thoroughly understand the composition and potential impacts of narratives used in mental health interventions. This can be accomplished by measures such as INCRESE, a 77-item tool designed to capture key narrative elements (3). INCRESE was recently used in the Narrative Experiences Online (NEON) intervention study to tag narratives with various characteristics (2). This allowed participants to choose which narratives to engage with and to avoid narratives they anticipated might contain potentially triggering content. INCRESE was developed through a rigorous process that included a systematic review identifying three overarching categories—narrative form, structure, and content—comprising nine dimensions for example genre, emotional tone, trajectory and use of metaphor (13). This knowledge was used while creating INCRESE, alongside other research about recovery and mental health, including a theoretical framework for recovery narratives (16) and research on recovery factors (17). In characterizing a narrative, there is a risk of reducing it. To avoid this, INCRESE is used to condense narratives, that is to shorten them while preserving their core by identifying predefined recovery-related themes within a narrative. By doing so, INCRESE may assist individuals and practitioners in finding narratives that are helpful to them. In the development of INCRESE, moderate to perfect interrater and test-retest scores for included items were found. Coders found INCRESE both feasible and acceptable in terms of administrative burden (3). INCRESE has since been translated to Dutch (INCRESE-NL, 18). The results mirrored the ones observed in the English version. Please see **Table 1** for an overview of English INCRESE.

**Table 1:** Overview of english INCRESE.

Section	Description/examples of content within each section
1. Narrative Eligibility	Codes whether the narrative is a mental health recovery narrative as defined in the NEON study
2. Narrative Mode	Codes whether the narrative contains text, audio, images as well as length of the narrative
3. Narrator Characteristics	The demographics of the narrator and their stage of recovery, possible disability, and diagnosis
4. Narrative Characteristics	Genre, positioning towards mental health services, tone, relationship with recovery, and trajectory of the narrative
5. Content Warnings	Examples: Abuse or sexual violence, self-harm including eating disorder
6. Turning Points	Examples: Taking charge, rude awakening
7. Narrative Content	Examples: Family, peer support, psychological services

However, because most narratives used in the studies of both English INCRESE and INCRESE-NL were created by adults, the instrument may not fully capture the distinct characteristics of recovery narratives produced by young individuals and adolescents. Existing recovery frameworks such as CHIME (17) require adaptation for young individuals who navigate unique developmental processes (19). Young individuals’ dependence on caregivers (20, 21), the influence of peers and the school system (22), and their ongoing biological and social development (20) all shape their recovery trajectories. While the process of mood improvement may be the same for adults and youth, young individuals will not return to previous functioning but rather develop toward new levels of capability as they recover (23). At the same time, it is important not to conflate chronological age with expressive capacity. The ability to articulate recovery experiences may be influenced by multiple factors, including cognitive, linguistic, neuro- and social development, as well as individual and cultural differences. Consequently, differences in how recovery is expressed should not be attributed to age alone. As for Sámi adolescents, they are exposed to contextual factors that differ from those of the general population, indicating that their recovery narratives may include characteristics not considered in English INCRESE and that their experiences may not fit the original definition of recovery. When the CHIME framework has been examined through a cultural lens, it appears that although it seems to be applicable across many different cultures, some adaptations may be required. Among Indigenous populations, the notion of a collective identity, involving connections to both family and cultural heritage, has emerged as particularly important (24). The transmission of knowledge between generations has been identified as a source of meaning, and religion and spirituality appear to play a more influential role in non-Western than in Western cultures (24).

The NEON Young Norway study (nord.no/neonyoung) aims to enhance understanding of recovery narratives among young individuals aged 16–30 in Norway. Since the project is carried out in collaboration with a Norwegian municipality within the administrative area for Sámi languages, with recognized importance for South Sámi culture, we make specific efforts to include the narratives of Sámi youth. To analyze these narratives comprehensively, a Norwegian version of INCRESE is needed. As the English INCRESE was developed for adults, direct translation is insufficient. Instead, a contextually and developmentally adapted version is needed. The aims of this study were to translate English INCRESE into Norwegian, to adjust the inventory for Norwegian youth including both the general and Sámi populations, and to assess the reliability, acceptability and feasibility of INCRESE-Norwegian Young (NY).

## 2 Methods

### 2.1 Study design

We conducted the study in three stages

1. Translation of INCRESE from English into Norwegian
2. Contextual adaptation to Norwegian and Sámi youth contexts
3. Psychometric evaluation (reliability, feasibility, and acceptability).

### 2.2 Procedures

#### Stage 1: Translation

The translation work followed The UPSIDES (Using Peer Support in Developing Empowering Mental Health Services) Proportionate Translation Methodology (25). This methodology included preparation such as familiarization with the material and identifying appropriate translators. The next step was forward translation by two independent members of the research team fluent in English and native in Norwegian. Conceptual and cultural equivalence guided all translation decisions. After completion, the two versions were merged. Areas of divergence were identified in a reconciliation meeting involving both translators, the first author, and our representative from Youth Mental Health Norway (who was actively involved as a member of the research team throughout the project). When alternative translations were equally accurate, the shortest and most straightforward option was selected. The inventory was then back translated by a professional translator, and the back translation was reviewed by the first author and authors of the English INCRESE. The Norwegian translation was finalized and underwent psychometric evaluation complemented by qualitative analysis, which is the last prescribed step in the translation methodology.

#### Stage 2: Contextual adaptations

In conducting the contextual adaptations, we examined aspects of English INCRESE that needed to be adapted to fit the context of young Norwegians. Apart from considering differences, we also looked for aspects that may be important in Norwegian recovery narratives but that were not present in English INCRESE, and that we therefore needed to add. Given the narrative and context-dependent nature of recovery stories, the adaptations made were intended to enhance conceptual and contextual relevance rather than to constitute a formal test or cultural adaptation of the inventory. Adaptations and/or additions were conducted with regards to general Norwegian conditions, to fit the age of our target group (16–30 years) and to be inclusive to Sámi youth.

To adapt the translation to Norwegian conditions, a panel with three researchers and our representative from Youth Mental Health Norway reviewed items that might differ in meaning between English and Norwegian contexts. Adjustments were identified where cultural norms, service structures, or terminology required modification.

To refine the translation for the target population of young people, our Lived Experience Advisory Panel (LEAP), which at the time comprised five young Norwegians with lived experience of mental health challenges and/or Sámi background reviewed English INCRESE, focusing on the content warnings and the content section. They reviewed item by item to identify missing content relevant to youth recovery narratives, assess items that were not applicable to young individuals and to ensure age-appropriate language and concepts.

To ensure inclusivity for young Sámi, the inventory was reviewed in collaboration with our Sámi LEAP member, our project partners from a Sámi municipality and with representatives from the Sámi Norwegian National Advisory Unit on Mental Health and Substance Use​ (SANKS). The Sámi contributors could identify problematic items, propose new constructs and reject categories from the original inventory. All suggestions were incorporated in INCRESE-NY. Additions focused on cultural practices, and elements known to shape Sámi youth experiences.

Feedback from all consultations was integrated and incorporated into the revisions of the items.

#### Stage 3: Psychometric evaluation

Sample size was calculated using an online calculator (https://wnarifin.github.io/ssc/ssicc.html) with the following parameters: minimum reliability (ρ0 = 0.40), expected reliability (ρ1 = 0.65), significance level (α=0.05), power (80%), four coders and no expected drop-out rate. The power calculation determined 32 narratives as the minimum required sample size. Although the power calculation was based on four coders, we included five coders in the study to ensure higher data quality and to safeguard the coding process, for example, in case one coder became unavailable due to illness or other unforeseen circumstances.

Before initiating their independent coding, the coders participated in a two-session workshop. The first session provided an overview of INCRESE-NY and coding instructions, as well as an overview of the NEON Young Norway study context. Coders then independently coded five narratives. Two weeks later, coders met for a second session where discrepancies were discussed. Discussion of discrepancies revealed that most differences stemmed from unclear instructions, which were subsequently clarified. INCRESE-NY (final version) can be found in the **Supplementary Material**. There, all items and coding instructions for each item can be found. Generally, verbal and manifest content is coded, and many items include the instruction to avoid interpretation.

The recovery narratives included were chosen to match the intended target group of the study (Norwegians aged 16-30). Age of the narrators spanned from 16-29, mean age 23.94 (*SD* = 3.52). Twelve narratives had been donated to the project, a further 20 consisted of stories publicly accessible online. Narrators varied in age, gender, and ethnicity, including six Sámi narrators. Narrative formats included written texts, songs, videos, podcast, interviews and comic strips. Some narratives were very brief (such as a song and a poem) and others were longer (e.g. videos and podcast episodes lasting almost an hour).

The five coders were members of the Neon Young Norway research team with diverse mental health-related professional backgrounds. Several coders also had lived experience of mental health challenges. Their age ranged from 27-44. Four were female, one male. Two of the coders were not native speakers of Norwegian but had solid familiarity with the language. One through long-term residence in Norway. One coder was a native Swedish speaker and the close linguistic relatedness of the Scandinavian languages enabled a high level of comprehension of Norwegian. All coders underwent the same training and participated in both reliability assessments.

Feasibility and acceptability were evaluated according to definitions by Proctor et al. (26) where acceptability is regarded as perceived agreeableness or satisfaction, and feasibility as the extent to which the instrument can successfully be used in practice. Data were collected: Three narrative-specific open-ended questions were completed after coding each narrative, addressing missing elements, reflections on the narrative, and reflections on INCRESE-NY. Further, after coding all narratives the coders answered a survey in which they rated the difficulty of each INCRESE-NY section on a 1–5 Likert scale (1 = very easy; 5 = very difficult). In the survey, we also asked for suggestions for improvements and about their experience coding the narratives. We tracked time to estimate administrative burden, including engagement and coding time.

### 2.3 Analysis

To assess test-retest reliability, we calculated Cohen’s kappa for each coder individually. An overall estimate was obtained by computing an item-based mean, giving each item equal weight. For each item, we calculated kappa value, 95% confidence intervals (via bootstrap resampling with 10,000 iterations), percentage agreement and standard deviation.

Interrater reliability was calculated using Gwet’s AC1 (27), along with percentage agreement for comparison.

We predefined kappa and AC1 thresholds above .40 as indicative of an acceptable level of agreement. Analyses were conducted in R 4.5.2 (28) and IBM SPSS Statistics 31.

To identify candidate refinements, the survey responses were analyzed using thematic analysis (29, 30). Given the brevity of responses, analysis remained at the semantic level. NVivo (version 15.3.2) was used for data organization.

## 3 Results

### Stage 1: Translation

The first author and authors of the English INCRESE reviewed the back translation and identified seven issues (three instances where a word had disappeared in the translation process, two where there still remained two alternative translations, one where the chosen word “hysteric” was considered sexist and therefore removed, and the word “short” had been translated as “shorter”). The word “distress” was translated either as emotional strain or difficulty, depending on context. Necessary corrections were made, yielding INCRESE-NY version 1, comprising 77 items.

### Stage 2: Contextual adaptation

Six items of INCRESE-NY version 1 were adjusted to match Norwegian professional, educational and cultural contexts. For example, British categories of mental health profession were replaced with Norwegian equivalents in the item “relationship with professionals”. Ethnicity categories were revised based on the Norwegian National Research Ethics Committees’ guidelines (Ethnic minorities | Forskningsetikk) and Statistics Norway’s classification of immigrants (https://www.ssb.no/befolkning/innvandrere/artikler/slik-definerer-ssb-innvandrere). Additionally, two changes were made to reflect Norwegian understanding of the concept of recovery: we added “according to how the individual defines it” to the definition of recovery and we changed the name of the category “diagnosis” to “diagnosis/coping problem”. Our LEAP reviewed INCRESE-NY and suggested eight changes (e.g. they strongly disagreed with categorizing self-harm and eating disorder as one content warning). To ensure the inventory’s acceptability to the target group, we incorporated all their suggested changes.

A total of 14 items were modified or added to better reflect young individuals’ experiences, for example “bullying” was added as an example under the item “education”. Additional items were included to capture youth specific challenges previously documented in research, such as “involuntary school absence”. The age categories were changed to fit our target group (16–30 years).

As an adaptation for Sámi Inclusion, Joik (chanting) was added as a narrative form relevant to Sámi traditions. Three additional items were also incorporated to more fully capture themes relevant to Sámi youth. For example, “reindeer herding” was added under the content section. We considered including Sápmi as a possible location of a narrator, but in consultation with a Sámi LEAP member we decided not to, since Sápmi is part of the other locations and many Sámi have left their traditional lands. Sámi was included as an ethnic category and in the instructions, it was made clear that it was possible to select multiple ethnicity categories, something that was important to our LEAP members. The resulting INCRESE-NY version 2 comprises 91 items and can be found in the **Supplementary Materials**. All adaptations to INCRESE-NY version 1 are shown in **Table 2**.

**Table 2:** Adaptations to INCRESE-NY version 1.

Section of INCRESE NY	Item name	Adaptation	Rationale
Introduction		Added: “according to how the individual defines it”	Adapted to fit the concept of recovery
	Audio	Added “Joik” (chanting) as an example	Adaptation to Sámi
2. Mode	Moving images	Added “animation” as an example	Adaptation to youth target group
	Static images	Changed “graphic novel” to “cartoon” in the examples	Adaptation to youth target group
	Age	Changed age categories	Adaptation to youth target group
	Ethnicity	Changed ethnicity categories	Adaptation to Norwegian context
	Location of narrator	Changed geographical categories	Adaptation to Norwegian context
	Disability: medical illness	Added “medical illness”	LEAP-based acceptability adaptation
3. Narrator Characteristics	“Diagnosis” became “Diagnosis/coping problem”	Changed category heading to “diagnosis/coping problem”	Adapted to fit the concept of recovery
	Diagnosis/coping problem: involuntary school absence	Added involuntary school absence	Adaptation to youth target group
	Diagnosis/coping problem: loneliness	Added Loneliness	Adaptation to youth target group
	Diagnosis/coping problem: bullying	Added bullying	Adaptation to youth target group
	Diagnosis/coping problem: sleep disorders	Added sleep disorders	Adaptation to youth target group
5. Content Warnings	Loss of life or endangerment to life	Removed “suicide or suicide attempt” from the description	LEAP-based acceptability adaptation
	“Self-harm including eating disorders” became “Self-harm, suicide or suicide attempt”	Removed “eating disorder” both from heading and description	LEAP-based acceptability adaptation
	Eating disorders	Added a separate item called “Eating disorders”	LEAP-based acceptability adaptation
7. Narrative content	“Family” became “Family and relatives”	Changed category heading to “Family and relatives”	Adaptation ta Sámi culture
	Education	Added the example “bullying”	Adaptation to youth target group
	Receiving a diagnosis	Added the positive example “feeling sense of community”	LEAP-based acceptability adaptation
	Relationship with professionals	Added specific Norwegian professions to description	Adaptation to Norwegian context
	Psychological services	1. Changed heading to “psychological treatment/psychotherapy” 2. added the example “music therapy”	1. Adaptation to Norwegian context 2. LEAP-based acceptability adaptation
	Being in natural environments	Added the example “forest and mountain hikes”	1. Adaptation to Norwegian context and LEAP-based acceptability adaptation
	Spiritual/religious activities	Added examples “Exclusion, e.g. loss of identity and community”	LEAP-based acceptability adaptation
	Gaming	Added Gaming	Adaptation to youth target group
	Norms	Added Norms	Adaptation to youth target group
	Identity and self concept	Added to the existing item “stigma”	Adaptation to youth target group
	Social Media	Added Social media	Adaptation to youth target group
	Climate	Added Climate	Adaptation to youth target group
	Urbanity vs Rurality	Added Urbanity vs Rurality	Adaptation to Norwegian context
	Growing up in several cultures	Added Growing up in Several Cultures	Adaptation to youth target group and adaptation to Sámi culture
	Reindeer Herding	Added Reindeer Herding	Adaptation to Sámi culture
	Learning to speak Sámi	Added Learning to speak Sámi	Adaptation to Sámi culture

### Stage 3: Psychometric evaluation

Interrater reliability and mean test-retest reliability, including item-level values and percentage agreement for INCRESE-NY version 2 are shown in **Table 3**. Coder-specific reliability estimates for each item are shown in **Supplementary Table 1**.

Test-retest reliability for INCRESE-NY version 2 was calculated for 82 of 91 items. It was not calculated for total words and total length, since these are continuous measures, and not for seven other items with no variability. Mean test–retest reliability (Cohen’s kappa) across all items was moderate, κ = 0.59 (*SD* = 0.27) ranging from -0.02 (below chance) to 1.00 (perfect agreement). Mean percentage agreement between timepoints was 90.7% (*SD* = 10.03), with item-level agreement ranging from 51.9% to 100.0%. 16 items of 82 (19.5%) scored below the predefined cutoff of κ = 0.41. Eight of these items were derived from the items constructed especially for INCRESE-NY, such as “loneliness” (item 33 in the section of coping problems) and gaming (item 84 in the narrative content section). All items falling below cutoff are shown in bold in **Table 3**.

**Table 3:** Test-retest reliability, interrater reliability and percentage agreements for INCRESE-NY version 2.

Test-retest reliability				Interrater reliability	
Item	n	Mean_kappa (95% CI)	Mean % agreement (*SD*)	AC1 (95% CI)	% agreement
1. Lived experience account?	0	NA^a^	100. 0 (0.0)	NA^a^	100.0
2. Is this a narrative?	2	1	100.0 (0.0)	0.98 (0.96-1.00)	93.8
3. Adversity	0	NA^a^	100.0 (0.0)	NA^a^	100.0
4. Success	0	NA^a^	100.0 (0.0)	0.99 (0.98-1.00)	96.9
5. Written elements	4	0.68 (0.37-1.00)	96.9 (5.4)	0.84 (0.72-0.94)	71.9
6. Sound elements	4	0.95 (0.90-1.00)	96.9 (3.1)	0.90 (0.80-0.98)	84.4
7. Moving images	5	0.97 (0.94-1.00)	99.4 (1.3)	0.99 (0.96-1.00)	96.9
8. Static images	5	0.81 (0.71-0.90)	93.9 (4.2)	0.86 (0.74-0.95)	78.1
9. Total words	5	NA^b^		NA^b^	
10. Total length (mins)	5	NA^b^		NA^b^	
11. Gender	5	0.96 (0.94-0.99)	96.9 (2.2)	0.95 (0.90-0.99)	100,0
12. Age	5	0.94 (0.91-0.98)	94.4 (6.0)	0.83 (0.74-0.90)	56.3
13. Ethnicity	5	0.78 (0.64-0.92)	89.4 (9.3)	0.86 (0.77-0.93)	65.6
14. Stage of recovery	5	0.66 (0.47-0.86)	86.3 (15.7)	0.85 (0.75-0.92)	62.5
15. Location of narrator	5	0.80 (0.73-0.84)	87.6 (6.3)	0.76 (0.67-0.84)	46.9
16. Sexuality	5	0.92 (0.83-1.00)	97.5 (4.1)	0.91 (0.84-0.97)	81.3
17. Visual difficulty	1	**0.00 (0.00-0.00)**	98.8 (1.7)	0.98 (0.95-1.00)	96.9
18. Hearing difficuliy	0	NA^a^	100.0 (0.0)	NA^a^	100.0
19. Mobility difficultiy	4	1.00 (1.00-1.00)	99.4 (1.4)	0.99 (0.97-1.00)	96.9
20. Cognitive difficulty	2	0.47 (0.47-0.47)	98.2 (2.7)	0.97 (0.91-1.00)	93.8
21. Somatic illness	3	**0.22 (0.00-0.65)**	98.2 (1.7)	0.99 (0.97-1.00)	96.9
22. Self-care difficulties	2	**0.00 (0.00-0.00)**	97.6 (2.5)	0.97 (0.93-0.99)	87.5
23. Neuro-developmental	5	1.00 (1.00-1.00)	100.0 (0.0)	1.00 (1.00-1.00)	100.0
24. Eating related	5	0.89 (0.83-0.95)	96.3 (2.6)	0.94 (0.86-1.00)	90.6
25. Mood related	5	0.75 (0.67-0.84)	86.9 (7.2)	0.65 (0.51-0.78)	50.0
26. Personality related	0	NA^a^	100.0 (0.0)	NA^a^	100.0
27.OCD related	5	0.76 (0.58-0.93)	97.5 (2.6)	0.98 (0.96-1.00)	93.8
28. Psychosis related	3	**-0.02 (-0.03-0.00)**	98.2 (2.7)	0.99 (0.98-1.00)	96.9
29. Trauma related	5	0.68 (0.54-0.85)	95.7 (3.6)	0.96 (0.91-1.00)	90.6
30. Substance related	3	0.43 (0.00-0.65)	98.8 (1.7)	0.98 (0.93-1.00)	93.8
31. Non-diagnostic	4	**0.35 (0.23-0.48)**	88.2 (15.1)	0.89 (0.81-0.95)	65.6
32. School absence	4	0.55 (0.10-1.00)	93.2 (6.7)	0.92 (0.84-0.98)	84.4
33. Loneliness	5	**0.33 (0.27-0.41)**	82.0 (9.6)	0.64 (0.47-0.79)	43.8
34. Bullying	4	0.68 (0.47-0.89)	97.6 (2.5)	0.96 (0.90-0.99)	87.5
35. Sleep related	4	**0.36 (0.00-0.75)**	93.2 (6.8)	0.90 (0.82-0.97)	75.0
36. Genre	5	0.42 (0.31-0.55)	58.1 (13.7)	**0.36 (0.25-0.47)**	3.1
37. Positioning	5	0.92 (0.87-0.95)	93.8 (4.4)	0.85 (0.75-0.94)	71.9
38. Tone	5	0.63 (0.49-0.74)	80.6 (14.9)	0.67 (0.53-0.79)	46.9
39. Relation recovery	5	0.48 (0.29-0.65)	65.0 (15.0)	0.60 (0.51-0.70)	18.8
40. Trajectory	5	0.48 (0.44-0.52)	79.4 (5.7)	0.74 (0.64-0.84)	43.8
41. Metaphors	5	0.57 (0.41-0.77)	81.8 (12.4)	0.51 (0.35-0.68)	28.1
42. Abuse or sexual	5	0.82 (0.77-0.91)	97.6 (2.5)	0.96 (0.91-0.99)	87.5
43. Death or threatened death	3	**0.26 (0.00-0.78)**	95.7 (1.6)	0.94 (0.87-0.98)	84.4
44. Self injury	5	0.69 (0.62-0.81)	92.6 (3.5)	0.87 (0.75-0.96)	78.1
45. Violence	5	0.72 (0.54-0.91)	95.7 (3.5)	0.94 (0.86-0.99)	87.5
46. Discrimination	4	0.70 (0.61-0.78)	95.1 (3.5)	0.91 (0.83-0.97)	78.1
47. Eating disorder	5	0.91 (0.86-0.96)	96.9 (2.2)	0.97 (0.91-1.00)	93.8
48. Taking control	5	0.53 (0.39-0.63)	76.4 (8.3)	0.56 (0.41-0.71)	34.4
49. Support	5	0.62 (0.57-0.66)	82.4 (5.2)	0.63 (0.50-0.78)	46.9
50. Self-acceptance	4	0.46 (0.16-0.63)	85.0 (12.2)	0.56 (0.43-0.69)	18.8
51. Spiritual	4	0.48 (-0.03-1.00)	98.8 (2.7)	0.99 (0.97-1.00)	96.9
52. Rude awakening	5	0.62 (0.50-0.75)	94.4 (2.6)	0.90 (0.80-0.97)	81.3
53. Shift in identity	4	0.42 (0.12-0.70)	97.5 (2.6)	0.90 (0.81-0.97)	78.1
54. Pregnancy	0	NA^a^	100.0 (0.0)	NA^a^	100.0
55. Family	5	0.58 (0.54-0.62)	70.0 (4.2)	0.58 (0.48-0.71)	31.3
56. Care system	5	1.00 (1.00-1.00)	97.5 (5.6)	0.98 (0.94-1.00)	90.6
57. Education	5	0.53 (0.44-0.62)	69.9 (9.6)	0.62 (0.50-0.74)	37.5
58. Friendship	5	0.56 (0.46-0.66)	73.1 (9.4)	0.62 (0.51-0.75)	34.4
59. Relationships	5	0.83 (0.79-0.87)	93.2 (3.5)	0.90 (0.82-0.96)	75.0
60. Housing	5	**0.28 (0.00-0.57)**	85.7 (10.3)	0.89 (0.80-0.96)	75.0
61. Income	4	**0.40 (0.09-0.63)**	90.7 (7.4)	0.90 (0.81-0.98)	81.3
62. Work	5	0.69 (0.55-0.78)	85.7 (11.6)	0.68 (0.54-0.80)	43.8
63. Criminal justice	4	**0.33 (0.00-0.75)**	96.3 (2.6)	0.97 (0.91-1.00)	93.8
64. Diagnosis	5	0.71 (0.63-0.82)	84.3 (8.8)	0.76 (0.65-0.86)	53.1
65. Medication	5	0.62 (0.44-0.80)	87.5 (8.0)	0.84 (0.74-0.93)	71.9
66. Relation carer	5	0.67 (0.64-0.72)	84.3 (5.0)	0.74 (0.63-0.84)	50.0
67. Peer support	3	0.48 (0.00-0.79)	96.3 (1.4)	0.95 (0.90-0.99)	84.4
68. Involuntary MH use	5	0.95 (0.84-1.00)	98.8 (1.7)	0.96 (0.91-1.00)	90.6
69. Hospitalisation	5	0.87 (0.70-1.00)	95.6 (4.2)	0.95 (0.89-0.99)	87.5
70. Psychotherapy	5	0.60 (0.56-0.68)	77.5 (5.1)	0.70 (0.55-0.81)	46.9
71. Alternative therapies	0	NA^a^	100.0 (0.0)	NA^a^	100.0
72. Nature	5	0.75 (0.61-0.87)	88.2 (10.2)	0.83 (0.73-0.92)	65.6
73. Animals/pets	3	0.44 (0.00-0.75)	96.9 (3.1)	0.92 (0.83-0.98)	84.4
74. Community spaces	2	**0.08 (0.00-0.16)**	91.3 (10.7)	0.95 (0.89-0.98)	84.4
75. Hobbies	5	0.67 (0.57-0.78)	86.4 (7.9)	0.82 (0.71-0.90)	62.5
76. Physical activities	5	0.46 (0.34-0.59)	83.7 (9.7)	0.84 (0.75-0.92)	65.6
77. Activisme	5	0.62 (0.53-0.71)	84.4 (9.4)	0.76 (0.65-0.86)	50.0
78. Spiritual activities	5	1.00 (1.00-1.00)	99.4 (1.4)	1.00 (1.00-1.00)	100.0
79. Stigma	5	**0.36 (0.15-0.54)**	61.9 (22.4)	0.47 (0.36-0.59)	9.4
80. Caregiving responsibilities	2	0.49 (0.49-0.49)	99.4 (1.3)	0.99 (0.98-1.00)	96.9
81. Family history of MH issues	5	0.84 (0.76-0.92)	95.0 (4.8)	0.94 (0.88-0.98)	84.4
82. Diet/nutrition	5	0.76 (0.61-0.90)	93.2 (5.1)	0.84 (0.72-0.92)	65.6
83. Volunteering	4	**0.37 (0.08-0.65)**	90.7 (9.7)	0.92 (0.86-0.96)	71.9
84. Gaming	3	**0.33 (0.00-1.00)**	98.8 (1.7)	0.97 (0.92-1.00)	93.8
85. Social norms	5	**0.21 (0.07-0.35)**	81.8 (13.3)	0.82 (0.73-0.91)	59.4
86. Social media	5	0.44 (0.31-0.58)	81.9 (9.2)	0.85 (0.77-0.93)	65.6
87. Climate change	5	0.72 (0.48-0.96)	96.3 (2.6)	0.94 (0.86-1.00)	90.6
88. Urban–Rural Contexts	5	0.57 (0.51-0.62)	88.8 (3.6)	0.86 (0.76-0.94)	75.0
89. Raised in several cultures	4	0.56 (0.25-0.75)	88.8 (6.9)	0.92 (0.83-0.98)	84.4
90. Reindeer herding	5	0.77 (0.75-0.79)	97.5 (1.4)	0.97 (0.91-1.00)	93.8
91. Learning to speak Sámi	2	0.69 (0.53-0.84)	95.0 (5.7)	0.94 (0.88-0.99)	87.5

NA^a^= No variability. NA^b^ = Continuous measure.Values below the cutoff value of 0.41 are indicated in boldface.

Interrater reliability was calculated for 83 of 91 items. It was not calculated for total words and total length, since these are continuous measures, and not for six other items with no variability. Generally, inter-rater reliability across items was high, with a mean Gwet’s AC1 of 0.86 (*SD* = 0.14), indicating an almost perfect agreement. AC1 values ranged from 0.36 (fair) to 1.00 (perfect). The mean percentage agreement between the raters was 72.8% (*SD* = 26.64). One item (“Genre”) of 83 (1.2%) fell below the predefined acceptable level (shown in bold in **Table 3**). Using a more conservative threshold (AC1 ≥ 0.60) reduced the number of items meeting the agreement criterion by five (6/83, 7.2%).

In relation to feasibility, coders rated ease of coding for each section of INCRESE-NY version 2, as shown in **Table 4**.

**Table 4:** Coder rating of coding difficulty for each section of INCRESE-NY version 2.

Section	Mean rating (sd)	Interpretation
1. Narrative eligibility	1.40 (0.55)	Very easy
2. Narrative mode	1.40 (0.55)	Very easy
3. Narrator characteristics	2.20 (0.84)	Easy
4. Narrative characteristics	4.40 (0.55)	Difficult
5. Content warnings	2.40 (1.67)	Easy
6. Turning points	3.20 (0.84)	Neutral
7. Narrative content	2.40 (1.14)	Easy

Survey responses indicated that all narrative themes present in the material were not fully captured by the INCRESE-NY coding categories. Coders reported additional themes including influence of role models, openness in sharing one’s story, behavioral addictions, grief, and experiences of domestic violence and growing up with a parent with mental health or substance use problems. “I feel that the issue of neglect/an alcoholic caregiver does not fit many of the categories…” Coder 4 commented. “There are some things I am missing. For example, behavioral addiction such as gambling”, Coder 3 stated.

Coders noted that some instructions were unclear. For one thing, it was hard to know when to add a content warning or not. “I find the coding description for Section 5 (content warning) unclear; I feel like I code this differently every time”, Coder 2 reported after coding a certain narrative. In the survey conducted after finishing all coding, the coder elaborated this further:

Section 5: The coding instructions here are ambiguous because they say ‘Err on the side of caution, that is, tick all items that may apply,’ while under each theme it says ‘tick for direct experiences with…’. This made me very uncertain about when I should mark a content warning and when I should not.”

The instructions in Section 7 were also described as difficult to apply because, as Coder 1 stated “It is difficult to decide what is a significant part of the narrative”. Coder 2 provides insight into the challenges they have faced:

Section 7: It is difficult to assess what constitutes a significant part of the story when so many things are mentioned … I initially ticked off ‘relationships,’ then removed it, and then added it again … I tried to think about what the coding is intended to be used for. If it is to inform people who are trying to understand the content of the story, then this story is not really about relationships. But there were other things mentioned in less detail, so I left it. I find this very difficult.”

Coders reported that feasibility varied depending on the narrative format. The instrument was easiest to apply to long, text-based narratives “It is as if it were made for this type of narrative”, Coder 1 stated after coding a long text. However, multimedia or abstract formats, such as songs, poems, and artistic pieces, were perceived as more challenging to code systematically. Coder 5 elaborates:

I find songs difficult to evaluate. I have chosen to assume that the person is singing about themselves and their own experiences … It is also not clear whom she is referring to, whether it is family, friendship, or a romantic relationship.

Coders generally reported a positive experience engaging with the narratives and described the work as meaningful and valuable: “Some of the stories are genuinely interesting and fascinating to read. Combined with the stories that ‘end well,’ this makes the coding a generally pleasant experience”, Coder 4 stated. It could also provide valuable insights: “(I) gained better insight into how certain conditions and illnesses can affect individuals differently, and how their own perception of their conditions is shaped”, Coder 3 noted. The coders, however, described some narratives as emotionally challenging and expressed interest in a more structured system for emotional support. Coder 5 stated:

I felt that the coding process was very lonely and isolating, despite the heavy themes involved and the idea that we would be able to support one another. I also think it might have been wise to have more meetings among us coders.

Section 4 (Narrative Characteristics) was the least favored part of the instrument: “Section 4 was very difficult (IMPOSSIBLE in some cases) to code, in my opinion … Particularly difficult were: ‘tone,’ ‘genre,’ and ‘developmental trajectory.” (Coder 2). Coder 4 also describes genre as a particularly difficult item: “I still find ‘genre’ difficult. In particular, “endeavor” and “endurance” are difficult to distinguish”.

The mean time spent engaging with the narrative, was 19.03 minutes (SD = 4.04), with a wide range (5–65 minutes) depending on narrative format and complexity.

## 4 Discussion

This study translated, adapted, and preliminary evaluated INCRESE-NY for use with recovery narratives created by young Norwegians, including Sámi youth. Overall, the findings suggest that INCRESE-NY demonstrates adequate reliability, feasibility, and acceptability. However, reliability varied between items and the coders observed clear needs for refinement. With further refinement, INCRESE-NY appears to be a promising tool for characterizing youth recovery narratives in Norway. This may support young people and practitioners to make informed choices about which narratives to use in supporting recovery. Our results align with previous findings from the English INCRESE (3) and the Dutch INCRESE-NL (18). The findings provide preliminary support for the applicability of several INCRESE-NY to Norwegian and Sámi youth narratives following substantial contextual and cultural adaptation.

### 4.1 Translation and adaptation

The translation followed the UPSIDES Proportionate Translation Methodology and the rigorous methodology ensure a strong Norwegian version. Since INCRESE is an inventory for characterizing recovery narratives rather than a patient-reported outcome measure, formal guidelines for cultural adaptation of measurement instruments such as those proposed by Cruchinho et al. (31) were not directly applicable. Future research may build on this work by examining the applicability of INCRESE-NY across additional youth contexts within and beyond Norway, and, where relevant, by complementing the present approach with more formalized adaptation procedures.

While the present study focused on linguistic and contextual adaptation, broader conceptual issues warrant further consideration. Indigenous cultural orientations and practices are linked to wellbeing and health through strong cultural identities, traditional forms of teaching, storytelling traditions, and local understandings of self and personhood (11, 24, 32). Such factors may shape how recovery is understood and communicated. Future studies should therefore explore whether additional adaptations are required to support conceptual equivalence and to assess the extent to which the framework reflects Sámi ways of knowing and communicating.

One challenge encountered during the adaptation process concerned item reduction, as it was not possible to conclusively determine that certain characteristics would never occur in a recovery narrative. As a result, decisions favored retention rather than exclusion, leading to an expansion of the inventory from 77 to 91 items. While this enhanced the instrument’s coverage, it also increased its length and administrative burden. Future studies of INCRESE-NY could therefore systematically examine the frequency and utility of individual items across larger samples, with the aim of identifying candidates for potential reduction. An alternative approach may involve reconsidering the structure of the “Narrative Content” section, which currently comprises 38 items, for example by replacing it with a free-text field allowing narrators or coders to indicate the primary thematic content of the narrative. Such an approach could preserve sensitivity to narrative diversity while reducing item burden.

### 4.2 Reliability

Interrater reliability consistently showed higher values than test-retest reliability, with 16 (17.6%) items falling below the predefined cutoff. This finding should, however, be interpreted cautiously, as Gwet’s AC1 generally yields higher and more stable estimates than Cohen’s kappa when variability between ratings is limited (33). It is also noteworthy that percentage agreement was higher for the test-retest assessment (90.7%) than for the interrater reliability assessment (72.8%). This apparent discrepancy highlights the importance of considering chance-corrected agreement measures alongside percentage agreement. While percentage agreement reflects the proportion of identical ratings, it does not account for agreement occurring by chance and may therefore overestimate reliability when responses are concentrated in a limited number of categories. Conversely, kappa is known to be sensitive to prevalence and skewed category distributions, which can result in relatively low kappa values despite high observed agreement. Gwet’s AC1 typically produces higher estimates than kappa under such conditions, as it adjusts for chance agreement in a manner that is less affected by prevalence effects. The threshold of.41 for both kappa and AC1 values was set in accordance with the recommendations by Landis and Koch (34) and was consistent with the cutoff values used in the evaluation of both the English INCRESE (3) and INCRESE-NL (18). As no universally accepted AC1-specific cutoff values currently exist, the same threshold was applied to facilitate comparison across language versions of the instrument. Although a sensitivity analysis using a more conservative AC1 threshold of 0.60 classified five additional items as being below the agreement criterion, the overall conclusions remained unchanged, suggesting that the findings were reasonably robust to the choice of threshold.

Eight of the items with reliability values below the cutoff pertained to the newly added items (e.g., gaming and loneliness). As these items were developed specifically for the present adaptation, they may require further refinement and clearer operational definitions to improve coding consistency. No similar concentration of low-performing items was observed among the remaining domains, suggesting that reliability challenges were not confined to any particular conceptual area of the inventory. Although the coders described section four (Narrative Characteristics) as difficult to code, reliability values were generally acceptable for this section. The only exception is “genre”, where the interrater reliability was below cutoff. An item such as “genre” require subjective interpretation and pertain to abstract narrative qualities. An element like this is inherently difficult to code consistently and may complicate the use of interrater reliability (35), especially when the formats were as varied as in our study.

### 4.3 Feasibility

Feasibility ratings showed that most sections were easy or moderately difficult to code. However, Section 4 (Narrative Characteristics) emerged as the most challenging, replicating challenges reported in the Dutch translation (18). Items related to “genre” were particularly problematic and consistently disliked by coders. Narrative format had a notable impact on the results. INCRESE-NY worked best with long, text-based narratives, but was more difficult to apply to multimedia, artistic, and abstract formats such as songs, films, or poems.

Coder feedback revealed narrative elements not represented in the instrument. These omissions reflect broader developmental and contextual factors that influence youth recovery. Notably, the family system plays a central role in youth mental health (20, 21), and future versions of INCRESE-NY would benefit from fuller coverage of these themes. The identification of these gaps is a strength of the current study.

Expanding the “diagnosis” category to “diagnosis/coping problem” was conceptually aligned with recovery approaches but created overlap across sections. The same narrative element might be coded as a coping problem (Section 3), a turning point (Section 6), and narrative content (Section 7).

### 4.4 Acceptability

Coders generally found the work meaningful and acceptable. Nevertheless, engaging with multiple RNs, including some emotionally intense stories, was described as both rewarding and demanding. This underscores the need for information about reactions such as secondary traumatic stress (36) and on structured emotional support when coding sensitive narratives.

Mean administrative burden was 19 minutes, somewhat longer than the 15minute threshold used in the development of the English INCRESE. However, direct comparison is not possible because our instructions explicitly included time spent engaging in the narrative itself. Future studies should distinguish between time spent engaging with the narrative and time spent coding it. The increased coding time may also reflect the larger number of items in INCRESE-NY, further supporting the need to consider item reduction or restructuring of the inventory in future research.

Another aspect to consider while comparing time frames is the number of coded narratives. In the development of English INCRESE, 95 narratives were coded, whereas 32 narratives were coded in this study. It is reasonable to assume that the time spent on each narrative goes down as the coders grow more experienced with the coding procedure. In sum, given the wide variation in narrative length and format, ranging from short poems to an hourlong film, the observed timeframe appears reasonable.

### 4.5 Conceptual considerations regarding recovery

Qualitative feedback suggested that coder challenges arose from several distinct sources. Some difficulties appeared procedural in nature and may be addressed through clearer instructions, additional examples, or enhanced coder training. These included uncertainty regarding the use of content warnings and the interpretation of coding instructions. Other challenges appeared conceptual, as coders questioned how to determine what constitutes a significant component of a narrative, how narrative genres should be distinguished, and whether certain categories overlapped or were sufficiently comprehensive. Such concerns may reflect ambiguities within the framework itself rather than shortcomings in coder training.

Additional challenges may reflect cultural and developmental variation. Adolescence is not merely a chronological age category, and capacities relevant to narrative expression, including emotional regulation, self-reflection, and meaning-making, develop unevenly across adolescence and emerging adulthood. Such challenges may therefore reflect not only limitations of the coding framework but also developmental differences in young people’s ability to recognize, interpret, and communicate recovery-related experiences. INCRESE-NY was difficult to apply to multimedia formats, a limitation that may affect its ability to capture culturally embedded forms of expression such as music, ritual, storytelling, and visual art. These forms have been shown to be important for many young people, particularly those from culturally diverse or marginalized backgrounds (37). The observation that several recurring themes did not fit existing categories may indicate limits in content coverage rather than coder uncertainty.

Finally, some challenges may stem from inherent limitations of narrative data. Individuals may experience aspects of recovery that are difficult to articulate, and narratives may therefore provide only a partial representation of recovery processes. Consequently, disagreement may sometimes reflect limitations in what can be expressed and coded rather than weaknesses in the coding procedure itself.

Taken together, these findings suggest that some disagreements may stem not only from implementation challenges but also from the need for further refinement of underlying constructs and greater attention to cultural and developmental diversity. The feedback provided by the coders offers valuable insights that may inform future efforts to evaluate, refine, and expand the recovery framework underpinning INCRESE-NY.

### 4.6 Methodological strengths and limitations

A key limitation is that all coders were members of the research team. Although this may reduce variability in interpretations, the coders’ backgrounds (mental health professionals and individuals with lived experience) mirror those of likely future users, enhancing external validity. Still, future studies should involve external coders to evaluate generalizability. It is also unclear whether the fact that two of the coders were not native Norwegian speakers affected their coding. However, both reported having a good command of Norwegian, and no problems with comprehension were noted. Another limitation is that only two coders were within the same age range as the target group, and none were Sámi. For future research, it would be helpful to include younger coders to ensure that the interpretation of narratives is not dependent on age. Sámi coders should be included in order to assess cultural appropriateness. Beyond the coders, a larger narrative sample including more Sámi narratives should be used in future work with INCRESE-NY.

The sample narratives upon which the codings were based were produced by narrators aged 16-29. Although consistent with the target group of the Neon Young Norway project, this is a large age span. It is possible that the narratives differ between younger and older individuals within the age group, and that INCRESE-NY performs differently depending on age of the narrator. In this study, the sample size did not allow for subgroups analyses based on the age of the narrator, which must be considered a limitation of the study.

It is also important to acknowledge that individuals may experience aspects of recovery that are not readily articulated in ways that can be captured by INCRESE-NY. The absence of codable content should therefore not be interpreted as the absence of recovery-related experiences, but rather as an indication that such experiences were not expressed in a way that could be identified within the inventory’s coding framework. However, this limitation should be considered in light of the intended purpose of INCRESE-NY. The instrument is designed to assess recovery elements that are explicitly expressed in narratives and are therefore accessible to a recipient other than the narrator. Consequently, the focus on manifest content is not only a methodological choice but also reflects the context in which the instrument is intended to be used.

Although this paper includes elements intended to capture features of Sámi recovery narratives, the assumptions behind INCRESE-NY is based on an individualistic definition of recovery. It does not consider family and community relationships, belonging, cultural continuity, collective agency and participation in shared practices as recovery processes, aspects that have proven to be important in indigenous populations (24). Although the inventory has been adapted to be inclusive to the narratives of Sámi youth, it’s foundation on majority assumptions of health and recovery must be regarded as a limitation. While the present adaptation process sought to increase the cultural relevance of INCRESE-NY for use with Sámi narratives, this should not be taken to imply that the concept of recovery itself is necessarily understood in identical ways across cultural contexts. Recovery has largely been developed within Western mental health traditions, and further research is needed to explore the extent to which its underlying assumptions align with Sámi perspectives on health, well-being, identity, and healing. Consequently, the present study should be understood as an adaptation of an existing recovery-oriented framework rather than a validation of the recovery construct within a Sámi worldview. Future research should assume a bottom-up perspective, and, with the starting point in Sámi recovery narratives, take a new look at the construct of recovery itself.

The involvement of the LEAP throughout the adaptation process is a major strength. Their experiential knowledge informed the adaptation process and contributed to making INCRESE-NY more developmentally and contextually relevant for young Norwegians, including Sámi youth. Another strength lies in the rigorous procedures applied to translation and contextual adaptation.

## 5 Conclusions

The findings suggest that INCRESE-NY is a promising instrument for characterizing recovery narratives created by young people in Norway and may have potential applications in research, clinical, and peer-support contexts. By facilitating a more systematic examination of narrative features, the instrument may support the informed and responsible use of recovery narratives in youth mental health. Although overall reliability was acceptable, reliability varied considerably between items, and the evaluation identified several areas for further refinement, including clarification of newly added items, more detailed guidance for coding the abstract Section 4, clearer boundaries between overlapping categories, and possible expansion of content domains to better reflect the diversity and complexity of young people’s recovery experiences. Future research should examine conceptual and cultural equivalence, developmental differences in narrative expression, and the representation of relational and non-verbal dimensions of recovery. Continued iterative development, in collaboration with young people and cultural experts, may further strengthen the inventory’s sensitivity, relevance, and practical utility.

## Data Availability

The raw data supporting the conclusions of this article will be made available by the authors, without undue reservation.
