# Supplementary material for: Translation, contextual adaptation, and preliminary evaluation of INCRESE-Norwegian Young
Source: Front Psychiatry. 2026 Oct 14;17:1893622. doi: 10.3389/fpsyt.2026.1893622 (PMC13732449; doi:10.3389/fpsyt.2026.1893622)
Supplement: Supplementary file 1 [file SupplementaryFile1.docx]

**Hva er INCRESE-NY?**

«Inventory of Characteristics of Recovery Stories- Norwegian Young (INCRESE-NY)» er et instrument for å karakterisere recoveryhistorier som handler om å bli bedre fra eller mestre psykiske plager. Begrepet «recovery» defineres som «en måte å leve et meningsfullt og tilfredsstillende liv på (slik individet selv opplever det), med eller uten psykiske plager. INCRESE oppsummerer spesifikke kjennetegn til en recoveryhistorie. Fokus er på selve historien og dens forteller, og det skal ikke være en vurdering av historiens kvalitet eller verdi.

**Hvem kan bruke INCRESE?**

INCRESE kan brukes av alle, men det er mer pålitelig om instrumentet/verktøyet brukes av kodere med personlig og/eller profesjonell erfaring med psykiske plager og recovery. Kodingens pålitelighet forbedres ved (a) å øve på koding av flere historier og at (b) to kodere uavhengig av hverandre fullfører INCRESE på historiene for å vurdere samsvar og diskutere uenigheter. Å bruke INCRESE innebærer å engasjere seg i recoveryhistorier knyttet til psykisk helse, hvor noen kan forårsake emosjonelt ubehag. Vi anbefaler å bruke tilstrekkelig tid til koding, og planlegge på forhånd tilnærminger som ivaretar egenomsorg og emosjonell støtte hos koderne.

**Hvem utviklet INCRESE?**

Den originale versjonen av INCRESE ble utviklet i England, som en del av NEON programmet som var finansiert av NIHR (referanse: RP-PG-0615-20016). Alle seksjoner kan modifiseres for å øke kulturell relevans. Henvisning til INCRESE:

Llewellyn-Beardsley J, Barbic S, Rennick-Egglestone S, Ng F, Roe J, Hui A, et al. INCRESE: development of an inventory to characterize recorded mental health recovery narratives. J Recovery Ment Health. (2020) 3:25–44. <https://jps.library.utoronto.ca/index.php/rmh/article/view/34626>

INCRESE-NY ble utviklet i Norge som en del av *The NEON Young Norway Study* som var finansiert av Nord universitet og Norges forskningsråd. Henvisning til INCRESE-NY:

**Instruksjoner for utfylling av INCRESE**

- Les instruksjonene for hver seksjon nøye, de skiller seg ut

For hvert element:

- Hvis avkryssingsboksen er tom (f.eks. element 1), kryss av hvis element gjelder for historien og la være blank om elementet ikke gjelder historien Hvis avkryssingsboksen spør om en mengde (kun element 9 og 10), skriv inn ditt estimat i avkryssingsboksen
- Hvis avkryssingsboksen har kategorier (f.eks. element 11), ring rundt den relevante kategorien

Hvis det ikke er mulig å krysse av et element, la avkryssingsboksen stå tom.

| Detaljer om historien og koder | |
| --- | --- |
| Historiens ID nummer |  |
| Tittel på historie (om dette finnes) |  |
| Dato for koding | **/ /** |
| Navn på koder |  |
| Tid brukt på å kode historien |  |

**SEKSJON 1: VURDERING OM HISTORIEN ER EN RECOVERYHISTORIE**

**Instruksjoner for utfylling av seksjon 1:** Denne seksjonen koder hvorvidt historien kan forstås som en psykisk helse recoveryhistorie som definert i NEON-studien. For målene med NEON- studien, er en historie om gjenoppretting av psykisk helse en recoveryhistorie, hvis alle fire punktene i seksjon 1 er krysset av. Denne delen er valgfri, og kan endres om nødvendig. I NEON, kodet vi seksjoner 2 til 7 bare hvis alle fire elementene i seksjon 1 ble krysset av.

| **#** | **Element** | **Beskrivelse** | **Svar**  **(kryss av alle som passer)** |
| --- | --- | --- | --- |
| **1** | Er dette en historie om levd erfaring? | Representerer fortellerens virkelige opplevelse av levde erfaringer med psykiske plager, det vil si deres egen opplevelse av psykisk helse. Historier presentert i tredje person er akseptable hvis de (identifiserbart) handler om fortellerens egne virkelige opplevelser for eksempel gjennom en tilhørende forfatterbiografi eller introduksjon. Ikke kryss av hvis historien handler om å støtte noen andre f.eks., fra perspektivet til et familiemedlems eller fagperson. Kryss av for alle historier kun med bilder. |  |
| **2** | Er dette en recoveryhistorie? | Representerer en serie av hendelser eller handlinger som skjer over en tidsperiode. Ikke kryss av om bidraget hovedsakelig er f.eks. et argument, diskusjon eller beskrivelse av noe (f.eks. en lidelse). For abstrakte historier f.eks. bildebaserte, dikt eller sanger, kryss av hvis historien inneholder identifiserbare sekvensielle detaljer eller en indikasjon på overgang. |  |
| **3** | Inneholder historien elementer av motgang eller utfordringer? | Historien inneholder forfatterdefinerte opplevelser av motgang eller utfordringer i forhold til psykiske plager. Disse aspektene kan variere fra en veldig liten del til en veldig stor del av historien. Motgang eller utfordring kan ta mange former, og kan ha blitt opplevd over kortere eller lengre perioder. |  |
| **4** | Inneholder historien elementer av suksess, styrker eller overlevelse? | Historiene inneholder forfatterdefinerte opplevelser av suksess, styrke eller overlevelse i forhold til psykiske plager. Disse aspektene kan variere fra en veldig liten del til en veldig stor del av historien. Suksess, styrker eller overlevelse kan ta mange former og kan ha blitt opplevd over korte eller lange perioder. |  |

**SEKSJON 2: HISTORIENS FORM**

**Instruksjoner for utfylling av seksjon 2:** Historiene kan være en blanding av moduser, eksempelvis inneholde både bilder og lyd (f.eks. film), eller den kan inneholde både bilde og tekst (f.eks. et kunstverk med tilhørende forfatterbiografi). Kryss av for hvert element som er til stede.

| **#** | **Element** | **Beskrivelse** | **Svar**  **(Kryss av alle som passer)** |
| --- | --- | --- | --- |
| **5** | Inneholder historien tekst? | Historien inneholder skriftlige elementer, f.eks. prosa, poesi, memoarer, bildetekst, tekstbaserte forfatterdetaljer eller biografi som følger med en bilde- eller videobasert historie. |  |
| **6** | Inneholder historien lyd? | Historien inneholder lydbasert elementer, f.eks. tale, sang, podkast, joik. |  |
| **7** | Inneholder historien bilder i bevegelser? | Historien inneholder bevegelige bilder, f.eks. film/video, animasjon. |  |
| **8** | Inneholder historien statiske bilder? | Historien inneholder statiske bilder som ikke beveger seg, f.eks. kunst, tegneserie, fotografi, photovoice, fotografier av forfatter med skrevet tekst. |  |
| **9** | Tekstens totallengde | Oppgi den totale lengden (i antall ord) av enhver form av tekst i historien. Vi foreslår å kopiere og lime inn teksten i f.eks. Word hvis mulig, be Chat GPT å estimere antall ord, ellers estimer selv. La boksen stå tom hvis fortellingen ikke inneholder tekst. | **Antall ord:** |
| **10** | Totallengde på lydfil eller video | Oppgi den totale lengden (i minutter) på alle av lyd- eller videoelementer. La stå tomt hvis fortellingen kun inneholder tekst eller statiske bilder. | **Antall minutter:** |

**SEKSJON 3: HISTORIEFORTELLERENS KARAKTERISTIKKER/KJENNETEGN**

**Instruksjoner for utfylling av seksjon 3:** For element 11 til 16: velg et svar hvis (1) fortelleren eksplisitt refererer til eller identifiserer det svaret ELLER (2) historien kommer fra en samling fra denne spesifikke gruppen ELLER (3) hvis ingen spesifikk referanse i verken historien eller samling, gjør ditt beste estimat. La avkrysningsboksen være blank hvis det ikke er mulig å komme med et estimat, for eksempel i noen bildebaserte historier.

For element 16-34: velg et spesifikt svar KUN hvis fortelleren eksplisitt identifiserer det svaret ELLER hvis historien kommer fra en samling fra denne spesifikke gruppen. La det være blankt hvis det ikke er spesifikt identifisert av fortelleren, dvs. **unngå å gjøre antakelser**.

| **#** | **Element** | **Beskrivelse** | **Svar**  **(Ring rundt ett per element)** |
| --- | --- | --- | --- |
| **11** | Kjønn | Svar ”annet” hvis fortelleren identifiserer seg som f.eks. trans, transkjønnet, ikke-binær eller annen kjønnsidentitet. Om ingenting nevnes, utgå fra fortellerens navn. | Gutt/Mann |
|  |  |  | Jente/Kvinne |
|  |  |  | Annet: |
|  |  |  | Ønsker ikke svare |
| **12** | Alder |  | 16-19 |
|  |  |  | 20-24 |
|  |  |  | 25-30 |
| **13** | Etnisitet |  | Norsk |
|  |  |  | Innvandrer |
|  |  |  | Norskfødt med en innvandrerforelder |
|  |  | F eks samisk. | Samisk (Urfolk) |
|  |  | Jøder, kvener, rom, romanifolket og skogfinner. | Nasjonal minoritet |
|  |  |  | Annet: |
|  |  |  | Ønsker ikke svare |
| **14** | Stadiet av recovery | Moratorium/ foreløpig stadium: ikke for øyeblikket engasjert i å tenke på eller arbeide mot recovery eller velvære. | Tenker ikke ennå på recovery |
|  |  | Bevissthet/ettertanke/forberedelsesstadium: gjenoppvåkning av håp, bevissthet om avhengighet, tatt beslutning om å gjenoppbygge livet. | Tenker på recovery |
|  |  | Gjenoppbygging/handling/opprettholdelse og vekststadium: aktivt engasjement, gjenoppbygging av livet, leve utover funksjonsnedsettelse. | Arbeider med recovery |
|  |  | Fortelleren avviser eksplisitt konseptet “recovery" slik som det brukes innen psykisk helsetjenestene. | Avviser recovery |
| **15** | Lokasjon for forteller | Velg bosted der de fleste område som majoriteten av hendelsene finner sted i historien. | Nord-Norge |
|  |  |  | Trøndelag |
|  |  |  | Vestlandet |
|  |  |  | Østlandet |
|  |  |  | Sørlandet |
|  |  |  | Utenfor Norge |
|  |  |  | Ønsker ikke svare |
| **16** | Seksuell legning | La avkryssingsboksen være blank hvis seksualitet ikke er identifisert av fortelleren, **dvs**. **unngå å gjøre antakelser**. | Biseksuell |
|  |  |  | Homofil mann |
|  |  |  | Heteroseksuell |
|  |  |  | Lesbisk/homofil kvinne |
|  |  |  | Annet: |
|  | **Funksjonsnedsettelse** | Kryss av hvis fortelleren refererer til å ha **betydelige og langvarige** vansker på grunn av psykiske eller fysiske plager. Psykiske plager er ikke inkludert som en kategori fordi alle fortellinger per definisjon vil inkludere disse. La være blank hvis ingen funksjonsnedsettelse er identifisert av fortelleren, **dvs. unngå å gjøre antakelser**. | **Svar**  **(kryss av alle som passer)** |
| **17** | Synsvansker | Blindhet eller delvis syn. Ikke kryss av dersom det kun er basert på at fortelleren bruker briller. |  |
| **18** | Hørselsvansker | Døvhet eller nedsatt hørsel. |  |
| **19** | Bevegelsesvansker /utholdenhetsvansker | Vanskeligheter med å f.eks. gå, klatre trapper, løfte og bære ELLER nedsatt utholdenhet eller pustevansker. |  |
| **20** | Kognitive vansker | Læringsvansker (f.eks. for eksempel kognitive funksjonsnedsettelser), huske (f.eks. på grunn av demens), eller konsentrasjon. |  |
| **21** | Somatisk sykdom | F.eks. diabetes, astma, kreft, hjertefeil, medfødt variasjon, kroniske tilstander. |  |
| **22** | Vansker med egenomsorg | Vansker med egenomsorg slik som å vaske seg eller kle på seg. |  |
|  | **Diagnose/ Mestringsproblem** | For element 23-35, kryss av hvis fortelleren identifiserer kategorien som sin diagnose/mestringsproblem. Ikke kryss av element 23-35 hvis fortelleren identifiserer hva som har blitt anvendt på dem, men som personen avviser. Hvis ingen kategori er identifisert OG punkt 31 ikke gjelder, la element 23-35 stå tomme. | **Svar**  **(Kryss av alle som passer)** |
| **23** | Nevro-utviklingsrelatert | F.eks. autismespektrum (ASD), Aspergers syndrom, ADHD. |  |
| **24** | Mat- kropp- og/eller vektrelatert | F.eks. anoreksi, overspising, bulimi. Problematikk kring mat, kropp, vekt og trening. |  |
| **25** | Humørrelatert | F.eks. angst, bipolar lidelse 1 og 2, cyklotymi, depresjon, dystymi, maniske episoder, panikkanfall, fobier, fødselsdepresjon. |  |
| **26** | Personlighetsrelatert | F.eks. emosjonell ustabil personlighetsforstyrrelse (BPD), narsissistisk personlighetsforstyrrelse, paranoid personlighetsforstyrrelse. |  |
| **27** | Tvangsrelatert | F.eks. tvangslidelse (OCD), kroppsdysmorfisk lidelse. |  |
| **28** | Schizofreni eller annen psykoserelatert | F.eks. schizofreni, vrangforestillinger, schizoaffektiv lidelse, hører stemmer. |  |
| **29** | Traume-/stressrelatert | F.eks. tilpasningsforstyrrelse, akutt stresslidelse, posttraumatisk stresslidelse (PTSD). |  |
| **30** | Rusrelatert | F.eks. problematisk bruk av alkohol, cannabispreparater, kokain, andre sentralstimulerende midler, opioider, hallusinogener, sedative, løsemidler (sniffe lim/ bensin etc.). |  |
| **31** | Bruker et ikke-diagnostisk rammeverk | Avviser **aktivt** bruk av diagnose ELLER bruker bevisst andre begreper i stedet for å referere til diagnose, slik som å høre stemmer, traumebasert belastning, å være en overlever av tjenester, identifiserer seg selv som gal, ELLER bruker en alternativ forklaringsramme, slik som åndelig krise. Ikke kryss av element 31 bare fordi element 23 til -30 ikke er krysset av. |  |
| **32** | Ufrivillig skolefravær | Isolasjon, manglende kontakt med jevnaldrende, frykt for å gå til utdanningsstedet sitt. |  |
| **33** | Ensomhet | Utenforskap, isolasjon, forringet livskvalitet, økt indre stress/uro. |  |
| **34** | Mobbing | Fysisk og/eller psykisk. |  |
| **35** | Søvnvansker | F.eks. vanskelig å sovne, hyppige oppvåkninger, våkner for tidlig, sove for mye eller utfordringer knyttet til å koble av ”stenge ut sosiale medier”, være pålogget. |  |

**SEKSJON 4: HISTORIENS KARAKTERISTIKKER/KJENNETEGN**

**Instruksjoner for utfylling av Seksjon 4:** Mange historier kan passe til mer enn ett av disse svarene. Velg for hvert element det svaret som passer best til historien totalt sett.

| **#** | | **Element** | | **Beskrivelse** | | **Svar**  **(ring rundt et av alternativene per element)** | |
| --- | --- | --- | --- | --- | --- | --- | --- |
| **36** | | Sjanger | | Hovedfokuset er på fortellerens flukt fra eller motstand mot faktorer som er identifisert som hindrer for deres recovery, f.eks. vanskelige familiære eller andre omstendigheter, skadelige eller unyttige hjelpetjenester, former for urettferdighet, uenighet i omstridt diagnose, avvisning av diagnostisk rammeverk. | | Flukt | |
|  |  |  |  | Hovedfokuset er på fortellerens overlevelse og evne til å fortsette til tross for langvarige forhold som for eksempel emosjonell belastning, tap, traumer, eller vanskelige omstendigheter. Omstendighetene kan være pågående eller i fortiden. | | Utholdenhet | |
|  |  |  |  | Hovedfokuset er på fortellerens måter å opprettholde sin bedring eller oppnå velvære på, f.eks., aktiviteter/ holdninger/læring/relasjoner som hjelper, måter å håndtere eller gjenopprette orden på, prestasjoner eller måloppnåelse. | | Anstrengelse | |
|  |  |  |  | Hovedfokuset er på fortellerens transformasjon av deres forståelse eller perspektiv på deres situasjon, f.eks. finne et nytt forklarende rammeverk; deres egen selvstyrking eller selvrealisering; en reisesøken eller reise av utforsking; en følelse av forløsning gjennom noe større enn seg selv, enten menneskelig eller åndelig. | | Opplysning | |
| **37** | | Plassering | | Psykiske helsetjenester er stort sett beskrevet som positive faktorer i fortellerens recovery f.eks. gjennom behandlinger eller tjenester som fungerte, å ha positive relasjoner med psykisk helsearbeidere, eller gjennom å levere tjenester (f.eks. Ansettelse som likeperson/erfaringskonsulent). | | Innenfor helsetjenester | |
|  |  |  |  | Psykiske helsetjenester/behandling er stort sett beskrevet som negative faktorer i fortellerens recovery, som aktivt forhindrer bedring eller forverrer psykiske plager, f.eks. krenkende behandling, negative holdninger hos personalet, tap av rettigheter eller verdighet. | | På tross av helsetjenester | |
|  |  |  |  | Psykiske helsetjenester/behandling nevnes ikke, f.eks. forteller oppnådde recovery på andre måter, eller gjennom tilgang til alternative behandlinger/tjenester, eller de er kun minimalt omtalt. F. eks. fortelleren fant behandlinger/tjenester nøytrale eller ineffektive, men ikke skadelige. | | Utenfor helsetjenester | |
| **38** | | Tone | | Positive tilstander, f.eks. oppstemt, fornøyd, håpefull, stolt, optimistisk, reflekterende. | | Oppstemt | |
|  |  |  |  | Negative tilstander, f.eks. opprørt, unnskyldende, intens, pessimistisk, trist, rystet, selvkritisk. | | Nedstemt | |
|  |  |  |  | Provoserende eller stimulerende, f.eks. sint, utfordrende, trassig, protesterende. | | Kritisk | |
|  |  |  |  | Flat, f.eks. saklig, fratatt rettigheter, resignert, f.eks. 'slik er det bare'. | | Nøytral | |
| **39** | Forhold til recovery | | Psykiske plager er hovedsakelig beskrevet i fortiden, f.eks. fortelleren forventer ikke at vedkommende skal oppleve dem i fremtiden; snakker om «å være «frisk» /recovered». | | Recovered/ Frisk | |  |
|  |  |  | Recovery/opplevelse av velvære er beskrevet som veletablert, f.eks. Fortelleren opplever hovedsakelig velvære, har tiltro til at fremtidige psykiske plager vil være håndterbare. | | Lever godt | |  |
|  |  |  | Recovery/opplevelse av velvære er beskrevet som under forbedring, f.eks. opplever fortelleren velvære og også regelmessige tider med stress, er bekymret for at fremtidige psykiske plager kanskje ikke er håndterbare. | | Gjør fremskritt | |  |
|  |  |  | Recovery/opplevelse av velvære er beskrevet som utfordrende eller tentativ, f.eks. fortelleren er fokusert på å komme seg gjennom en situasjon, er utholdende i møte med svært vanskelige omstendigheter, forventer at psykiske plager vil fortsette i fremtiden. | | Overlever dag for dag | |  |
|  |  |  | Fortelleren avviser konseptet “recovery” slik det brukes i psykiske helsetjenester. | | Avviser recovery | |  |
| **40** | Utviklingsforløp | | Totalt sett har formen på fortellingen en gradvis fremgang mot recovery eller velvære. Fortellingen kan inneholde tilbakeslag eller perioder med vanskeligheter, men fokuset er på fremgang. | | Opp | |  |
|  |  |  | Totalt sett har formen på fortellingen både oppturer mot helse/velvære og nedturer mot vanskeligheter. Oppturer og nedturer kan være dramatiske/berg-og-dal-baneendringer eller mer langvarige over tid, men fortellingen er mer eller mindre jevnt fordelt mellom oppturer og nedturer. | | Opp og ned | |  |
|  |  |  | Totalt sett har formen på fortellingen ingen betydelige opp- eller nedturer, f.eks. kan fortelleren beskrive en følelse av å stagnere eller ta en dag av gangen. | | Horisontal | |  |
|  |  |  | Totalt sett blir formen på fortellingen avbrutt av en enkelt krise eller vanskeligheter før den gjenopptar sin tidligere form og retning, f.eks. opplever fortelleren en traumatisk hendelse eller ulykke etter hvilket livet vender tilbake til "normalt". | | Avbrutt | |  |
|  |  |  | Totalt sett er formen på historien preget av at den veksler mellom sekvenser av fortvilelse/vanskeligheter, gradvis bedring, periode med velvære før historien går tilbake til fortvilelse/vanskeligheter, hvor all tidligere følelse av recovery eller velvære er tapt eller fraværende. | | Sirkulær | |  |
| **41** | | Bruk av metafor eller symbolsk språk | | Hvis fortellingen inneholder tekst eller tale, brukes metaforisk eller symbolsk språk? Velg ja for alle bildebaserte fortellinger. | | Ja | |
|  |  |  |  |  |  | Nei | |

**SEKSJON 5: INNHOLDSADVARSLER**

**Instruksjoner for utfylling av seksjon 5:** Kryss av for alle elementene som nevnes i historien. Aspekter kan variere fra å være en veldig liten del (kort omtalenevnt) til en veldig stor del (hovedemnet) av historien. Eksemplene er ikke fullstendige. Feil på siden av forsiktighet, det vil si kryss av for alle elementene som kan gjelde. Ikke kryss av hvis en forteller kun refererer til uspesifiserte traumer, f.eks. «traumet jeg opplevde» eller «de skremmende hendelsene jeg opplevde».

|  | **Element** | Beskrivelse | Svar  (kryss av alle som passer) |
| --- | --- | --- | --- |
| 42 | Mishandling eller seksuell vold | Direkte opplevelser eller vitne til enhver form for seksuell, fysisk eller emosjonell mishandling, forsømmelse, partnervold/vold i hjemmet eller seksuelle voldshandlinger, inkludert f.eks. voldtekt eller voldtektsforsøk, seksuelle overgrep, kjønnslemlestelse, moderne slaveri, sexhandel, seksuell utnyttelse av barn, pornografisk utnyttelse eller å være vitne til seksuelle handlinger, ulovlig/upassende bruk av tvang, misbruk av medisiner (f.eks. oversedasjon), tvangsmating eller tilbakeholdelse av mat, påtvungen sosial isolasjon (hindre noen fra for eksempel å få tilgang til tjenester eller se venner), mobbing, tvang, nettmobbing, trakassering, ydmykelse, trusler, verbale overgrep. |  |
| **43** | Tap av liv eller fare for liv | Direkte opplevelse eller vitne til f.eks. innleggelse til intensivbehandling, sorg, diagnose av en livstruende tilstand, tap av graviditet, naturkatastrofe, alvorlig ulykke, terrorangrep, tortur, traumatisk fødsel, traumatisk avslutning av graviditet, voldelig død av en annen, bli truet med våpen, krig/militær kamp. |  |
| **44** | Selvskading, selvmord eller selvmordsforsøk | Direkte opplevelser eller vitne til f.eks. bevisst skade eller skade på seg selv, forsømmelse av seg selv, alkohol- eller rusmisbruk, selvmord eller selvmordsforsøk. |  |
| **45** | Vold eller aggresjon | Direkte opplevelser eller vitne til f.eks. aggresjonshandlinger, slagsmål og opptøyer. |  |
| **46** | Urettferdighet, fordommer og diskriminering | Direkte opplevelser, vitne til eller henvisning til f.eks. hatprat, fordommer eller diskriminerende handlinger/atferd/beslutninger grunnlag av f.eks. hudfarge, funksjonsnedsettelse, etnisk opprinnelse, kjønnsidentitet, nasjonalitet, rase, religion, og seksuell legning. Erfaringer kan være på individuelt/mellommenneskelig nivå, organisasjons-/institusjonsnivå (f.eks. psykisk helsetjenester, fengsler) eller på systematisk/strukturelt nivå (historiske, kulturelle, juridiske, politiske eller økonomiske systemer). |  |
| **47** | Spiseforstyrrelser | Spiseforstyrrelsesrelatert atferd. |  |

**SEKSJON 6: VENDEPUNKT**

**Instruksjoner for utfylling av seksjon 6:** Alle historier vil ikke ha et vendepunkt. Kryss av KUN hvis fortelleren identifiserer elementet som et vendepunkt, eller bruker lignende språk, f.eks. ‘etter X var ting annerledes/bedre/enklere’; øyeblikket/tiden/personen/boken/situasjonen/hendelsen som endret ting var X’; "beslutningen om å X var den største faktoren; «Jeg innså etter X at ting måtte endres». **La avkryssingsboksen stå tom hvis elementet ikke er til stede, eller er til stede, men ikke identifisert av fortelleren som et vendepunkt, dvs. det vil si unngå å gjøre antakelser**. La avkrysningsboksen stå tom hvis det ikke er mulig å krysse av noen elementer, f.eks. ved bildebaserte fortellinger.

| **Fortellingsinnhold relater til vendepunkter, det vil si avgjørende faktor(er) i recoveryprosessen.** | | | |
| --- | --- | --- | --- |
| **#** | **Element** | **Beskrivelse** | **Svar**  **(kryss av alle som passer)** |
| **48** | Ta kontroll | Endring etter å ha tatt kontroll, f.eks. over egen sykdom, bedringsprosess, problematisk rusbruk, eller eget liv generelt. Kan være plutselig og avgjørende eller en lengre prosess. Kan være å akseptere eller avvise hjelp eller behandling f.eks. å bestemme seg for å ta eller slutte med medisinering. |  |
| **49** | Intervensjoner/støtte fra andre | Endring etter f.eks. å ha fått tilgang til medisiner som virker, behandling, grupper eller tjenester (enten direkte relatert til psykisk helse eller ikke), mottatt støtte fra familie eller venner, bli konfrontert av familie eller venner. |  |
| **50** | Selv- aksept /modenhet | Endring etter f.eks. en økning i selvtillit, vekst i selvbevissthet, økt modenhet, emosjonell frigjøring, bevegelse bort fra internalisert stigma. Dette kan skje gjennom egen læring/indre arbeid, å bli eldre, involvering med støtte- eller andre grupper, eller rådgivning/terapi. |  |
| **51** | Åndelig/  eksistensiell opplevelse | Endring etter f.eks. å finne en følelse av mening/hensikt, opplevelse av et stort skifte i perspektiv, bli med i et åndelig/religiøst fellesskap, ha utført spirituelle praksiser, konverteringsopplevelser, drømmer, positive visjoner, bli bedt for, støtte fra en veileder/lærer. |  |
| **52** | “Røff oppvåkning” | Endring etter et sjokk eller å ha innsett hvor ille ting har blitt, f.eks. å bli innlagt på sykehus, bli flyttet til en langtidsavdeling, sett negative effekter på familiemedlemmer, dødsfallet til noen nære, et selvmordsforsøk. |  |
| **53** | Skifte i identitet | Endring etter å ha gjenvunnet en stigmatisert identitet f.eks. basert på etnisitet, kjønn, seksualitet, psykisk helsestatus. Dette kan skje gjennom å finne andre med lignende opplevelser, egen læring, eget indre arbeid eller å bli involvert i aktivisme. |  |

**SEKSJON 7: HISTORIENS INNHOLD**

**Instruksjoner for utfylling av seksjon 7:** Kryss av for alle emner som er en vesentlig del av historien. **Ikke ta med korte omtaler eller kun fakta utsagn**, for eksempel en kort omtale av medisiner. For emner som ikke presenteres som hovedsakelig positive eller hovedsakelig negative (for eksempel er fortelleren nøytral, eller har like positive og negative erfaringer), kryss av for Til stede. Hvert element skal enten være tomt (hvis emnet ikke er til stede i fortellingen) eller ha nøyaktig én celle avkrysset. Hvis historien bare uttrykker en abstrakt følelse (f.eks. noen bilder eller sanger) la stå tomt.

| # | Punkt | Beskrivelse | Til stede | Hovedsaklig positivt | Hovedsaklig negativt |
| --- | --- | --- | --- | --- | --- |
| 54 | Graviditet/fødsel | Positive eksempler: ønsket graviditet, graviditet som et positivt vendepunkt.  Negative eksempler: traumatiske svangerskap/fødselsopplevelser, uønsket graviditet, abort, vanskeligheter etter fødsel, vansker med å bli gravid, eller evnen til å få barn. |  |  |  |
| 55 | Slekt og familie | Positive eksempler: emosjonell eller praktisk støtte eller omsorg fra familiemedlemmer, forhold til slekten/familien som en kilde til lykke eller motstandskraft.  Negative eksempler: egen eller foreldres skilsmisse, separasjon, samlivsbrudd eller pågående konflikt, død eller tap i slekten/familien. |  |  |  |
| 56 | Å være under omsorg | Positive eller negative opplevelser av adopsjon, å leve i fosterhjem, eller omsorg utenfor foreldrene. |  |  |  |
| 57 | Utdanning | Positive eksempler: å ha positive muligheter for læring/opplæring, engasjere seg i selvutdanning/selvhjelp.  Negative eksempler: vanskeligheter i skolen/universitetet, ufrivillig skolefravær, skolefravær, mobbing, å bli utestengt, og manglende trivsel på skolen å bli pålagt aktiviteter. |  |  |  |
| 58 | Vennskap | Positive eksempler: støtte fra venner.  Negative eksempler: tap av vennskap, ensomhet, sosial isolasjon, død av en som står nær. |  |  |  |
| 59 | Forhold | Positive eksempler: støttende partner, nytt forhold som gir håp.  Negative eksempler: vansker eller konflikt med partner. |  |  |  |
| 60 | Bosituasjon | Positive eksempler: å ha et eget hjem, hjemmet som en kilde til stolthet eller fristed.  Negative eksempler: å være i en sårbar bosituasjon eller hjemløs. |  |  |  |
| 61 | Inntekt | Positive eksempler: inntekt som muliggjør valg, forbedring i inntekt/ velferdssystemet.  Negative eksempler: gjeld, økonomiske vanskeligheter, fattigdom, dårlig opplevelse av velferdssystemet. |  |  |  |
| 62 | Arbeid | Positive eksempler: gir mening og hensikt, utvikler en positiv identitet.  Negative eksempler: utbrenthet, arbeidsrelatert stress, ustabile arbeidsforhold, arbeidsledighet, uønsket pensjonering. |  |  |  |
| 63 | Rettssystemet | Positive eksempler: fengsel som gir trygghet, et vendepunkt eller muligheter.  Negative eksempler: å bli arrestert, være i fengsel eller en ungdomsinstitusjon, ha et familiemedlem som sitter i fengsel. |  |  |  |
| 64 | Diagnose | Positive eksempler: gir mening i en situasjon, er en lettelse, gir svar eller følelse av håp, følelse av fellesskap.  Negative eksempler: uenighet knyttet til diagnose, følelse av stigmatisering, tap av håp. |  |  |  |
| 65 | Medisinering/ medisinske intervensjoner | Positive eksempler: føle seg bedre, oppleve lettelse, ha håp.  Negative eksempler: føle seg verre, virket ikke, var ikke frivillig, hadde uønskede bivirkninger. |  |  |  |
| 66 | Relasjon til profesjonell hjelper | Positive eller negative opplevelser av relasjoner til for eksempel kommunalt psykisk helseteam arbeidere, kriseteam, helsesøster, psykiater, psykiatrisk sykepleier, psykolog, sosialarbeider, miljøterapeut i skolen, ungdomsarbeider, utekontakt. |  |  |  |
| 67 | Likepersonsstøtte | Positive eller negative opplevelser av å gi eller motta individuell eller gruppebasert likepersonstøtte. Kan være formell f.eks. innen psykisk helsetjeneste eller Recoveryskole, eller uformelle f.eks. fra støttegrupper som (ideelle/interesseorganisasjoner) eksempler: AA, YMHN, hjelpetelefoner og hjelpechatter. |  |  |  |
| 68 | Ufrivillig bruk av psykisk helsetjeneste | Positive eller negative opplevelser relater til tvangsinnleggelse på institusjon/sykehus, tvungen medisinering. |  |  |  |
| 69 | Sykehusinnleggelse | Positive eller negative opplevelser av frivillig eller ufrivillig psykiatrisk sykehusinnleggelse. |  |  |  |
| 70 | Psykologisk behandling/psykoterapi | Positive eller negative opplevelser av å få eller gi samtaleterapi eller rådgivning, som kunst eller kreativ terapi, musikk terapi, kognitiv atferdsterapi (CBT), dialektisk atferdsterapi (DBT), gruppebehandling, psykoterapi, personorientert, psykoanalytisk, psykodynamisk eller løsningsfokusert terapi. |  |  |  |
| 71 | Alternativ behandling/ healing | Positive eller negative opplevelser av f.eks. akupunktur, homeopati, massasje, meditasjon, Reiki, tradisjonell kinesisk medisin (TCM). |  |  |  |
| 72 | Å være i naturen | Positive eller negative opplevelser av å være i naturen/natur eller utendørsaktiviteter slik som skog- og fjellturer, utendørsterapi. |  |  |  |
| 73 | Dyr/kjæledyr | Positive eller negative opplevelser av: kontakt med dyr, inkludert servicehund, besøkshund eller omsorg for et kjæledyr. |  |  |  |
| 74 | Møteplasser i lokalsamfunnet | Positive eller negative opplevelser av: engasjere seg med lokalsamfunnet, enten i grupper eller organisasjoner uavhengig om de er psykisk helserelatert eller ikke. Sosiale møteplasser. |  |  |  |
| 75 | Hobbyer/interesser/kreative aktiviteter | Positive eller negative opplevelser av: fritidsaktiviteter, hobbyer, avkoblingsaktiviteter eller kreative aktiviteter f.eks. kunst, håndverk, joik, musikk (lytte eller spille), opptredener, lese, danse, skrive. |  |  |  |
| 76 | Fysiske aktiviteter | Positive eksempler: å opprettholde god fysisk form, delta i idrett eller andre fysiske aktiviteter.  Negative eksempler: å være ute av stand til å delta på slike aktiviteter, deltakelse som leder til at du føler deg verre. |  |  |  |
| 77 | Aktivisme/ engasjement | Positive eksempler: Deltakelse i politiske eller andre aktiviteter for å oppnå endring i en gruppe, organisasjon, tjeneste eller system.  Negative eksempler: Deltakelse i overnevnte aktiviteter som leder til f.eks. utmattelse, følelse av å bli overveldet. |  |  |  |
| 78 | Åndelige, religiøse aktiviteter og opplevelser | Positive eksempler: deltakelse i et religiøst/åndelig fellesskap, gruppe eller sted for tilbedelse, eller deltakelse i åndelige praksiser, f.eks. som bønn, meditasjon, og retreater. Varsel eller hjelp fra døde forfedre. Negative eksempler: ovennevnte åndelige aktiviteter/ opplevelser som oppleves som tvungne eller som bidrar til psykiske plager. Ekskludering, for eksempel tap av identitet og fellesskap. |  |  |  |
| 79 | Stigma/ identitet/ selvbilde | Positive eksempler: Å avvise eller overvinne stigma eller skam knyttet til psykiske plager eller av andre grunner, f.eks. etnisitet eller kjønn. Hensikt/finne seg selv.  Negative eksempler: å oppleve andres misbilligelse eller egen skam knyttet til psykiske plager eller andre årsaker. Tap av fellesskap og relasjoner. |  |  |  |
| 80 | Omsorgsansvar | Positive eksempler: omsorg for noen andre som gir en følelse av stolthet.  Negative eksempler: omsorg for noen andre som forverrer egne psykiske plager. |  |  |  |
| 81 | Familieerfaringer med psykiske plager | Positivt eksempel: psykiske plager hos andre familiemedlemmer fører til felles forståelse.  Negativt eksempel: psykiske plager hos andre familiemedlemmer har en skadelig effekt på fortellerens egen psykiske helse. |  |  |  |
| 82 | Kosthold/ernæring | Positive eller negative opplevelser av kosthold eller ernæring knyttet til å forbedre, opprettholde eller forverre fortellerens psykiske helse. |  |  |  |
| 83 | Frivillighet/ å hjelpe andre | Positive eller negative opplevelser av alt frivillig arbeid, dugnad. |  |  |  |
| 84 | Gaming | Positivt: Følelse av tilhørighet/fellesskap, mestringsfølelse, validering, følelsesmessig utløp, sosial eksponering.  Negativt: Sosial isolasjon, ensomhet, tap av mestring, frustrasjon, avhengighet, mobbing. |  |  |  |
| 85 | Normer | Positivt: Fellesskap, struktur, trygghet, stabilitet, samfunnsmessig anerkjennelse og inkludering.  Negativt: Tap av selv/identitet, polarisering, opplever misbilligelse og sosial avvisning når man utfordrer normer. |  |  |  |
| 86 | Sosiale medier | Positivt: Fellesskap og tilhørighet, selvfølelse, glede, deling, validering, kulturell eksponering, åpenhet.  Negativt: Tap av identitet og selv, sosial isolasjon, ekskludering, økt negativt samfunnspress f.eks. kroppsbilde, polarisering, kriminalitet, mobbing. |  |  |  |
| 87 | Klima | Positivt: Fellesskap gjennom et felles mål og tilhørighet, formål, håp.  Negativt: Frykt, håpløshet, maktløshet, tap av mening og stabilitet. |  |  |  |
| 88 | Urbanitet eller ruralitet | Ruralitet: positiv: fellesskap, kontekst. Negativ: vanskelig å skille seg ut, opplevelse av å bli observert.  Urbanitet: Positiv: rom for mer mangfoldighet, negativt: forsvinner i mengden. |  |  |  |
| 89 | Vokst opp i flere kulturer | Positiv: rik på erfaringer, økt kulturforståelse, kan ta det beste fra hver av kulturene, flerspråklig.  Negativt: krysspress, opplevelse av å være annerledes, ikke passe inn (verken i norsk eller de andre kulturene), identitetsproblemer, språkutfordringer, diskriminering/stigma. |  |  |  |
| 90 | Reindrift | Positiv: kunne delta i reindrift, være i miljøet.  Negativ: ikke mulig å delta i reindrift pga bosted, arbeid, livssituasjon, familieforhold (slekt/familie driver ikke med rein, eller slektsforhold gjør at du ikke er «velkommen»). |  |  |  |
| 91 | Lære å snakke samisk | Positiv: har tilgang til samiske språkmiljø/ opplæring gjennom familie, venner, opplæringsinstitusjoner.  Negativ: har ikke tilgang til samisk språkmiljø, ikke mulighet til å delta ved opplæringsinstitusjoner. |  |  |  |
