# Supplementary material for: Translation, contextual adaptation, and preliminary evaluation of INCRESE-Norwegian Young
Source: Front Psychiatry. 2026 Oct 14;17:1893622. doi: 10.3389/fpsyt.2026.1893622 (PMC13732449; doi:10.3389/fpsyt.2026.1893622)
Supplement: Supplementary file 2 [file Table1.docx]

| **Table 1, supplementary material**  *Test-retest reliability and percentual agreement for each coder* | | | | | | | | | | |
| --- | --- | --- | --- | --- | --- | --- | --- | --- | --- | --- |
|  | **Coder 1** | | **Coder 2** | | **Coder 3** | | **Coder 4** | | **Coder 5** | |
| **Item** | **Kappa (95% CI)** | **%** | **Kappa (95% CI)** | **%** | **Kappa (95% CI)** | **%** | **Kappa (95% CI)** | **%** | **Kappa (95% CI)** | **%** |
| 1. Lived experience account? | NA | 100.0 | NA | 100.0 | NA | 100.0 | NA | 100.0 | NA | 100.0 |
| 2. Is this a narrative? | NA | 100.0 | NA | 100.0 | NA | 100.0 | NA | 100.0 | 1.00 (1.00-1.00) | 100.0 |
| 3. Narrator defined adversity | NA | 100.0 | NA | 100.0 | NA | 100.0 | NA | 100.0 | NA | 100.0 |
| 4. Narrator defined success | NA | 100.0 | NA | 100.0 | NA | 100.0 | **0.00 (0.00-0.00)** | 96.9 | NA | 100.0 |
| 5. Written elements | NA | 100.0 | NA | 100.0 | 1.00 (1.00-1.00) | 100.0 | 0.93 (0.73-1.00) | 96.9 | **0.27 (-0.09-0.78)** | 87.5 |
| 6. Sound elements | NA | 100.0 | NA | 100.0 | 0.93 (0.78-1.00) | 96.9 | 0.87 (0.64-1.00) | 93.8 | 0.86 (0.64-1.00) | 93.8 |
| 7. Moving images | 0.93 (0.74-1.00) | 96.9 | NA | 100.0 | 1.00 (1.00-1.00) | 100.0 | 1.00 (1.00-1.00) | 100.0 | 1.00 (1.00-1.00) | 100.0 |
| 8. Static images | 0.69 (0.36-0.93) | 87.5 | 0.94 (0.80-1.00) | 96.9 | 0.93 (0.74-1.00) | 96.9 | 0.93 (0.78-1.00) | 96.9 | 0.80 (0.56-1.00) | 90.6 |
| 9. Total words | NA | NA | NA | NA | NA | NA | NA | NA | NA | NA |
| 10. Total length (mins) | NA | NA | NA | NA | NA | NA | NA | NA | NA | NA |
| 11. Gender | 1.00 (1.00-1.00) | 100.0 | 0.94 (0.80-1.00) | 96.9 | 0.94 (0.81-1.00) | 96.9 | 0.94 (0.80-1.00) | 93.8 | 0.94 (0.81-1.00) | 96.9 |
| 12. Age | 0.95 (0.83-1.00) | 96.9 |  | 100.0 | 0.89 (0.71-1.00) | 93.8 | 0.77 (0.57-0.95) | 84.4 | 0.94 (0.80-1.00) | 96.9 |
| 13. Ethnicity | 0.59 (0.34-0.80) | 78.1 | 0.88 (0.67-1.00) | 93.8 | 1.00 (1.00-1.00) | 100.0 | 0.66 (0.41-0.88) | 81.3 | 0.85 (0.62-1.00) | 93.8 |
| 14. Stage of recovery | 0.77 (0.35-1.00) | 93.8 | NA | 100.0 | 0.43 (-0.07-0.87) | 87.5 | **0.19 (-0.10-0.47)** | 59.4 | **0.35 (-0.08-1.00)** | 90.6 |
| 15. Location of narrator | 0.83 (0.66-1.00) | 90.6 | 0.86 (0.68-1.00) | 90.6 | 0.78 (0.59-0.95) | 84.4 | 0.91 (0.77-1.00) | 93.8 | 0.67 (0.42-0.87) | 78.1 |
| 16. Sexuality | 1.00 (1.00-1.00) | 100.0 | 0.91 (0.64-1.00) | 96.9 | 1.00 (1.00-1.00) | 100.0 | 1.00 (1.00-1.00) | 100.0 | 0.71 (0.37-1.00) | 90.6 |
| 17. Visual difficulties | **0.00 (0.00-0.00)** | 96.9 | NA | 100.0 | NA | 100.0 | **0.00 (0.00-0.00)** | 96.9 | NA | 100.0 |
| 18. Hearing difficulties | NA | 100.0 | NA | 100.0 | NA | 100.0 | NA | 100.0 | NA | 100.0 |
| 19. Mobility difficulties | 1.00 (1.00-1.00) | 100.0 | NA | 100.0 | 1.00 (1.00-1.00) | 100.0 | **0.00 (0.00-0.00)** | 96.9 | NA | 100.0 |
| 20. Cognitive difficulties | 0.47 (-0.07-1.00) | 93.8 | NA | 100.0 | NA | 100.0 | 0.65 (0.00-1.00) | 96.9 | NA | 100.0 |
| 21. Somatic illness | **0.00 (0.00-0.00)** | 96.9 | 0.65 (0.00-1.00) | 96.9 | **0.00 (0.00-0.00)** | 96.9 | 1.00 (1.00-1.00) | 100.0 | NA | 100.0 |
| 22. Self-care difficulties | **0.00 (0.00-0.00)** | 93.8 | NA | 100.0 | NA | 100.0 | 0.65 (0.00-1.00) | 96.9 | **0.00 (0.00-0.00)** | 96.9 |
| 23. Neuro-developmental | 1.00 (1.00-1.00) | 100.0 | NA | 100.0 | 1.00 (1.00-1.00) | 100.0 | 1.00 (1.00-1.00) | 100.0 | 1.00 (1.00-1.00) | 100.0 |
| 24. Eating or food related | 0.91 (0.69-1.00) | 96.9 | NA | 100.0 | 0.79 (0.43-1.00) | 93.8 | 0.91 (0.67-1.00) | 96.9 | 0.82 (0.48-1.00) | 93.8 |
| 25. Mood related | 0.82 (0.53-1.00) | 93.8 | 0.87 (0.68-1.00) | 93.8 | 0.64 (0.29-0.91) | 87.5 | 0.55 (0.23-0.81) | 78.1 | 0.61 (0.34-0.87) | 81.3 |
| 26. Personality related | NA | 100.0 | NA | 100.0 | NA | 100.0 | NA | 100.0 | NA | 100.0 |
| 27. Obsessive-compulsive related | 0.65 (0.00-1.00) | 96.9 | NA | 100.0 | 1.00 (1.00-1.00) | 100.0 | 0.65 (0.00-1.00) | 96.9 | 0.48 (0.00-1.00) | 93.8 |
| 28. Schizofrenia related | **-0,03 (-0.08-0.00)** | 93.8 | NA | 100.0 | NA | 100.0 | NA | 100.0 | **0.00 (0.00-0.00)** | 96.9 |
| 29. Trauma related | 0.65 (0.00-1.00) | 96.9 | 0.47 (-0.07-1.00) | 93.8 | 1.00 (1.00-1.00) | 100.0 | 0.78 (0.00-1.00) | 96.9 | 0.62 (0.00-1.00) | 90.6 |
| 30. Substance related | 0.65 (0.00-1.00) | 96.9 | NA | 100.0 | **0.00 (0.00-0.00)** | 96.9 | 1.00 (1.00-1.00) | 100.0 | NA | 100.0 |
| 31. Non-diagnostic framework | 0.48 (0.00-1.00) | 93.8 | **0.00 (0.00-0.00)** | 96.9 | **0.19 (-0.09-0.48)** | 62.5 | NA | 100.0 | **0.27 (-0.10-0.78)** | 87.5 |
| 32. Involuntary school absence | **0.00 (0.00-0.00)** | 90.6 | NA | 100.0 | **0.20 (-0.13-0.65)** | 84.4 | 0.71 (0.33-1.00) | 90.6 | 1.00 (1.00-1.00) | 100.0 |
| 33. Loneliness | **0.27 (-0.10-0.78)** | 87.5 | 0.47 (-0.07-1.00) | 93.8 | **0.26 (-0.10-0.64)** | 78.1 | 0.59 (0.27-0.86) | 81.3 | 0.40 (0.10-0.68) | 68.8 |
| 34. Bullying | 0.47 (-0.05-1.00) | 93.8 | NA | 100.0 | 1.00 (1.00-1.00) | 100.0 | 0.84 (0.17-1.00) | 96.9 | 0.78 (0.00-1.00) | 96.9 |
| 35. Sleep related | **0,00** | 96.9 | NA | 100.0 | **0.00 (0.00-0.00)** | 96.9 | 0.52 (0.06-0.85) | 84.4 | 0.43 (-0.08-0.87) | 87.5 |
| 36. Genre | 0.40 (0.17-.0.62) | 56.3 | 0.64 (0.38-0.85) | 78.1 | **0.23 (0.00-0.44)** | 53.1 | **0.15 (-0.10-0.37)** | 40.6 | 0.45 (0.19-0.67) | 62.5 |
| 37. Positioning | 0.95 (0.81-1.00) | 96.9 | 0.94 (0.81-1.00) | 96.9 | 0.94 (0.80-1.00) | 96.9 | 0.77 (0.57-0.94) | 87.5 | 0.83 (0.63-1.00) | 90.6 |
| 38. Tone | 0.71 (0.47-0.93) | 84.4 | 0.56 (0.27-0.82) | 78.1 | 0.78 (0.46-1.00) | 93.8 | **0.29 (0.03-0.53)** | 56.3 | **0.37 (0.00-1.00)** | 90.6 |
| 39. Relationship with recovery | 0.63 (0.32-0.84) | 78.1 | 0.68 (0.44-0.89) | 81.3 | **0.29 (-0.03-0.58)** | 65.6 | **0.32 (0.07-0.58)** | 53.1 | **0.16 (-0.13-0.41)** | 46.9 |
| 40. Trajectory | 0.47 (0.09-0.76) | 78.1 | 0.44 (0.09-0.73) | 78.1 | 0.44 (-0.07-0.84) | 87.5 | **-0.12 (-0.20—0.03)** | 71.9 | 0.57 (0.27-0.83) | 81.3 |
| 41. Metaphors | 0.56 (0.23-0.86) | 81.3 | 0.49 (0.17-0.80) | 75.0 | 0.93 (0.78-1.00) | 96.9 | **0.37 (0.00-0.87)** | 90.6 | **0.31 (-0.02-0.62)** | 65.6 |
| 42. Abuse or sexual violation | 0.77 (0.31-1.00) | 93.8 | NA | 100.0 | 0.78 (0.00-1.00) | 96.9 | 1.00 (1.00-1.00) | 100.0 | 0.78 (0.00-1.00) | 96.9 |
| 43. Death or threatened death | **0.00 (0.00-0.00)** | 93.8 | **0.00 (0.00-0.00)** | 96.9 | 0.78 (0.00-1.00) | 96.9 | **-0.03 (-0.08-0.00)** | 93.8 | **0.00 (0.00-0.00)** | 96.9 |
| 44. Self injury | 0.61 (0.00-1.00) | 90.6 | 0.63 (-0.04-1.00) | 93.8 | 0.67 (0.37-0.93) | 87.5 | 0.85 (0.60-1.00) | 93.8 | 0.92 (0.72-1.00) | 96.9 |
| 45. Violence | 0.48 (0.00-1.00) | 93.8 | NA | 100.0 | 0.78 (0.00-1.00) | 96.9 | **0.35 (-0.08-1.00)** | 90.6 | 0.87 (0.48-1.00) | 96.9 |
| 46. Discrimination | 0.61 (-0.05-1.00) | 90.6 | NA | 100.0 | 0.79 (0.39-1.00) | 93.8 | 0.84 (0.00-1.00) | 96.9 | 0.77 (0.37-1.00) | 93.8 |
| 47. Eating disorder | 0.91 (0.69-1.00) | 96.9 | 0.89 (0.60-1.00) | 96.9 | 0.82 (0.52-1.00) | 93.8 | 0.91 (0.68-1.00) | 96.9 | 1.00 (1.00-1.00) | 100.0 |
| 48. Taking control | 0.63 (0.22-0.92) | 87.5 | **0.28 (-0.08-0.61)** | 68.8 | 0.49 (0.15-0.75) | 75.0 | **0.32 (-0.02-0.64)** | 68.8 | 0.63 (0.34-0.87) | 81.3 |
| 49. Support from others | 0.67 (0.39-0.93) | 84.4 | 0.58 (0.33-0.81) | 78.1 | 0.63 (0.35-0.87) | 81.3 | 0.81 (0.58-1.00) | 90.6 | 0.54 (0.24-0.81) | 78.1 |
| 50. Self-acceptance | **0.00 (0.00-0.00)** | 96.9 | 0.64 (0.00-1.00) | 93.8 | 0.57 (0.18-0.86) | 84.4 | **0.29 (0.00-0.58)** | 65.6 | 0.63 (0.31-0.87) | 84.4 |
| 51. Spiritual | **-0.03 (-0.08-0.00)** | 93.8 | NA | 100.0 | 1.00 (1.00-1.00) | 100.0 | 1.00 | 100.0 | NA | 100.0 |
| 52. Rude awakening | 0.47 (-0.05-1.00) | 93.8 | 0.65 (0.00-1.00) | 96.9 | 0.64 (0.00-1.00) | 93.8 | **0.37 (0.00-1.00)** | 90.6 | 0.87 (0.48-1.00) | 96.9 |
| 53. Shift in identity | **0.00 (0.00-0.00)** | 96.9 | 0.48 (0.00-1.00) | 93.8 | 0.78 (0.00-0.00) | 96.9 | 1.00 (1.00-1.00) | 100.0 | 0.43 (-0.08-0.87) | 100.0 |
| 54. Pregnancy | NA | 100.0 | NA | 100.0 | NA | 100.0 | NA | 100.0 | NA | 100.0 |
| 55. Family | 0.62 (0.40-0.83) | 75.0 | 0.54 (0.29-0.76) | 71.9 | 0.60 (0.34-0.81) | 71.9 | 0.52 (0.29-0.73) | 65.6 | 0.52 (0.30-0.75) | 65.6 |
| 56. Care system | 1.00 (1.00-1.00) | 100.0 | NA | 100.0 | 1.00 (1.00-1.00) | 100.0 | 0.46 (-0.05-0.85) | 87.5 | 1.00 (1.00-1.00) | 100.0 |
| 57. Education | 0.42 (0.21-0.66) | 62.5 | 0.70 (0.45-0.92) | 84.4 | 0.59 (0.35-0.79) | 75.0 | 0.46 (0.23-0.68) | 62.5 | 0.52 (0.29-0.72) | 65.6 |
| 58. Friendship | 0.67 (0.40-0.93) | 84.4 | 0.44 (0.14-0.73) | 78.1 | 0.61 (0.36-0.81) | 75.0 | 0.53 (0.27-0.75) | 68.8 | 0.39 (0.18-0.63) | 59.4 |
| 59. Relationships | 0.88 (0.48-1.00) | 96.9 | 0.75 (0.38-1.00) | 93.8 | 0.81 (0.59-1.00) | 93.8 | 0.63 (0.32-0.91) | 87.5 | 0.84 (0.59-1.00) | 93.8 |
| 60. Housing | **-0.05 (-0.10-0.00)** | 87.5 | 0.48 (0.00-1.00) | 93.8 | **0.22 (-0.11-0.65)** | 84.4 | **0.36 (0.11-0.62)** | 68.8 | 0.80 (0.45-1.00) | 93.8 |
| 61. Income | 0.48 (0.03-1.00) | 93.8 | **0.00 (0.00-0.00)** | 96.9 | **-0,03 (-0.08-0.00)** | 90.6 | 0.45 (0.14-0.74) | 78.1 | 0.69 (0.00-1.00) | 93.8 |
| 62. Work | 0.77 (0.56-0.95) | 87.5 | 0.41 (-0.05-0.77) | 81.3 | 0.78 (0.56-1.00) | 90.6 | **0.39 (0.12-0.65)** | 68.8 | 0.73 (0.51-0.93) | 100.0 |
| 63. Criminal justice system | **0.00 (0.00-0.00)** | 96.9 | NA | 100.0 | **0.00 (0.00-0.00)** | 96.9 | 0.58 (0.00-1.00) | 93.8 | **0.32 (0.00-0.49)** | 93.8 |
| 64. Diagnosis | 0.62 (0.27-0.87) | 84.4 | 0.91 (0.72-1.00) | 96.9 | 0.67 (0.37-0.92) | 84.4 | 0.57 (0.30-0.79) | 71.9 | 0.75 (0.53-0.90) | 84.4 |
| 65. Medication | **0.38 (0.12-0.66)** | 81.3 | 0.84 (0.30-1.00) | 96.9 | 0.83 (0-54-1.00) | 93.8 | **0.21 (-0.12-0.56)** | 78.1 | 0.67 (0.35-0.91) | 87.5 |
| 66. Relationship with MH professional | 0.64 (0.38-0.87) | 81.3 | 0.76 (0.49-1.00) | 90.6 | 0.67 (0.40-0.89) | 84.4 | 0.81 (0.62-0.95) | 87.5 | 0.64 (0.40-0.85) | 78.1 |
| 67. Peer support | **0.00(0.00-0.00)** | 96.9 | **0.00 (0.00-0.00)** | 96.9 | 0.66 (0.00-1.00) | 96.9 | **-0.03 (-0.08-0.00)** | 93.8 | 0.79 (0.00-1.00) | 96.9 |
| 68. Involuntary use of MH services | 1.00 (1.00-1.00) | 100.0 | NA | 100.0 | 0.74 (0.48-1.00) | 96.9 | 0.86 (0.49-1.00) | 96.9 | 1.00 (1.00-1.00) | 100.0 |
| 69. Hospitalisation | 1.00 (1.00-1.00) | 100.0 | 0.58 (0.00-1.00) | 93.8 | 0.75 (0.38-1.00) | 93.8 | 0.64 (0.00-1.00) | 90.6 | 1.00 (1.00-1.00) | 100.0 |
| 70. Psychotherapy | 0.58 (0.23-0.85) | 81.3 | 0.55 (0.32-0.78) | 75.0 | 0.75 (0.55-0,91) | 84.4 | 0.50 (0.29-0.73) | 71.9 | 0.56 (0.29-0.80) | 75.0 |
| 71. Alternative therapies | NA | 100.0 | NA | 100.0 | NA | 100.0 | NA | 100.0 | NA | 100.0 |
| 72. Being in natural environments | 0.88 (0.65-1.00) | 96.9 | 0.72 (0.00-1.00) | 93.8 | 0.79 (0.43-1.00) | 93.8 | 0.58 (0.19-0.87) | 84.4 | 0.49 (0.23-0.73) | 71.9 |
| 73. Animals/pets | **0.00 (0.00-0.00)** | 96.9 | NA | 100.0 | 0.58 (0.00-1.00) | 93.8 | 1.00 (1.00-1.00) | 100.0 | 0.75 (0.37-1.00) | 93.8 |
| 74. Community spaces | NA | 100.0 | NA | 100.0 | **0.00 (0.00-0.00)** | 96.9 | **-0.06 (-0.12-0.00)** | 78.1 | **0.16 (-0.14-0.59)** | 81.3 |
| 75. Hobbies | 0.64 (0.25-0.91) | 87.5 | 0.87 (0.48-1.00) | 96.9 | 0.72 (0.40-0.93) | 87.5 | 0.69 (0.42-0.92) | 84.4 | 0.49 (0.21-0.76) | 75.0 |
| 76. Physical activities | **0.33 (0.00-0.65)** | 81.3 | 0.69 (0.00-1.00) | 93.8 | 0.57 (0.20-0.86) | 84.4 | 0.74 (0.40-1.00) | 90.6 | 0.40 (0.14-0.63) | 68.8 |
| 77. Activisme | 0.75 (0.37-1.00) | 93.8 | 0.55 (0.21-0.82) | 84.4 | 0.54 (0.32-0.76) | 75.0 | 0.80 (0.45-1.00) | 93.8 | 0.51 (0.22-0.75) | 75.0 |
| 78. Spiritual activities | 1.00 (1.00-1.00) | 100.0 | NA | 100.0 | 1.00 (1.00-1.00) | 100.0 | 0.66 (0.00-1.00) | 96.9 | 1.00 (1.00-1.00) | 100.0 |
| 79. Stigma | 0.57 (0.27-0.79) | 75.0 | 0.44 (0.18-0.69) | 75.0 | **0.22 (-0.03-0.47)** | 56.3 | **0.38 (0.00-0.69)** | 78.1 | **-0.01 (-0.20-0.20)** | 25.0 |
| 80. Caregiving responsibilities | 0.49 (0.47-0.49) | 96.9 | NA | 100.0 | NA | 100.0 | NA | 100.0 | NA | 100.0 |
| 81. Family history of MH issues | 0.87 (0.48-1.00) | 96.9 | 0.74 (0.48-1.00) | 96.9 | 1.00 (1.00-1.00) | 100.0 | **0.39 (-0.03-0.78)** | 87.5 | 0.72 (0.00-1.00) | 93.8 |
| 82. Diet/nutrition | 0.59 (0.00-1.00) | 90.6 | NA | 100.0 | 0.90 (0.67-1.00) | 96.9 | 0.76 (0.50-1.00) | 90.6 | 0.70 (0.43-0.92) | 87.5 |
| 83. Volunteering | 0.65 (0.00-1.00) | 96.9 | 0.00 (0.00-0.00) | 96.9 | **0.00 (0.00-0.00)** | 96.9 | **-0.07 (-0.14-0.00)** | 87.5 | **0.17 (-0.11-0.51)** | 75.0 |
| 84. Gaming | **0.00 (0.00-0.00)** | 96.9 | NA | 100.0 | 1.00 (1.00-1.00) | 100.0 | 0.49 (0.48-0.49) | 100.0 | **0.00 (0.00-0.00)** | 96.9 |
| 85. Social norms | **0.17 (-0.11-0.60**) | 81.3 | 0.48 (0.00-1.00) | 93.8 | **-0.02 (-0.05-0.00)** | 87.5 | **-0.04 (-0.09-0.00)** | 87.5 | **0.26 (0.00-0.53)** | 59.4 |
| 86. Social media | **0.29 (-0.08-0.66)** | 78.1 | 0.63 (0.00-1.00) | 90.6 | 0.63 (0.00-1.00) | 90.6 | **0.18 (-0.10-0.63)** | 81.3 | **0.37 (0.07-0.63)** | 68.8 |
| 87. Climate change | 1.00 (1.00-1.00) | 100.0 | 0.49 (0.47-0.49) | 96.9 | **0.31 (0.00-0.49)** | 93.8 | **0.32 (0.00-0.49)** | 93.8 | 0.78 (0.00-1.00) | 96.9 |
| 88. Urban–Rural Contexts | 0.63 (0.00-1.00) | 90.6 | 0.48 (-0.03-1.00) | 93.8 | 0.51 (0.00-0.84) | 87.5 | 0.59 (0.14-0.90) | 87.5 | 0.58 (0.27-0.82) | 84.4 |
| 89. Raised in several cultures | 0.75 (0.37-1.00) | 93.8 | **0.00 (0.00-0.00)** | 93.8 | **0.08 (-0.10-0.32)** | 81.3 | **0.35 (-0.08-0.72)** | 81.3 | 0.64 (-0.02-1.00) | 93.8 |
| 90. Reindeer herding | 0.79 (0.00-1.00) | 96.9 | 0.74 (0.48-1.00) | 96.9 | 0.79 (0.00-1.00) | 96.9 | 1.00 (1.00-1.00) | 100.0 | 0.74 (0.48-1.00) | 96.9 |
| 91. Learning to speak Sámi | NA | 100.0 | NA | 100.0 | 0.53 (0.00-1.00) | 90.6 | **0.29 (-0.04-0.49)** | 87.5 | 0.84 (0.00-1.00) | 96.9 |
| *Note.* NA = Not Applicable due to low variability | | | | | | | | | | |
